# The Early Secretory Pathway Is Crucial for Multiple Aspects of the Hepatitis C Virus Life Cycle

**DOI:** 10.1128/jvi.00180-23

**Published:** 2023-06-20

**Authors:** Kiran Avula, Bharati Singh, Subhashish Samantaray, Gulam Hussain Syed

**Affiliations:** a Institute of Life Sciences, Bhubaneswar, Odisha, India; b Regional Centre for Biotechnology, Faridabad, Delhi, India; University of Southern California

**Keywords:** COPII vesicle, ERGIC-53, endoplasmic reticulum exit sites, HCV entry, HCV secretion, SEC16A, TRK-fused gene, TFG, early secretory pathway, hepatitis C virus

## Abstract

Although most of the early events of the hepatitis C virus (HCV) life cycle are well characterized, our understanding of HCV egress is still unclear. Some reports implicate the conventional endoplasmic reticulum (ER)-Golgi route, while some propose noncanonical secretory routes. Initially, the envelopment of HCV nucleocapsid occurs by budding into the ER lumen. Subsequently, the HCV particle exit from the ER is assumed to be mediated by coat protein complex II (COPII) vesicles. COPII vesicle biogenesis also involves the recruitment of cargo to the site of vesicle biogenesis via interaction with COPII inner coat proteins. We investigated the modulation and the specific role of the individual components of the early secretory pathway in HCV egress. We observed that HCV inhibits cellular protein secretion and triggers the reorganization of the ER exit sites and ER-Golgi intermediate compartments (ERGIC). Gene-specific knockdown of the components of this pathway such as SEC16A, TFG, ERGIC-53, and COPII coat proteins demonstrated the functional significance of these components and the distinct role played by these proteins in various aspects of the HCV life cycle. SEC16A is essential for multiple steps in the HCV life cycle, whereas TFG is specifically involved in HCV egress and ERGIC-53 is crucial for HCV entry. Overall, our study establishes that the components of the early secretory pathway are essential for HCV propagation and emphasize the importance of the ER-Golgi secretory route in this process. Surprisingly, these components are also required for the early stages of the HCV life cycle due to their role in overall intracellular trafficking and homeostasis of the cellular endomembrane system.

**IMPORTANCE** The virus life cycle involves entry into the host, replication of the genome, assembly of infectious progeny, and their subsequent release. Different aspects of the HCV life cycle, including entry, genome replication, and assembly, are well characterized; however, our understanding of the HCV release is still not clear and subject to debate due to varied findings. Here, we attempted to address this controversy and enhance our understanding of HCV egress by evaluating the role of the different components of the early secretory pathway in the HCV life cycle. To our surprise, we found that the components of the early secretory pathway are not only essential for HCV release but also contribute to many other earlier events of the HCV life cycle. This study emphasizes the importance of the early secretory pathway for the establishment of productive HCV infection in hepatocytes.

## INTRODUCTION

As per recent global estimation, around 58 million people have chronic hepatitis C virus (HCV) infection, and about 1.5 million new infections are reported annually (1). The newly developed direct-acting antivirals are effective at curing more than 95% of people with hepatitis C (2); however, these antivirals are expensive and less accessible. In 2019, around 3,00,000 people died due to HCV-associated end-stage liver disease, suggesting that public health challenges still remain (1). Due to the lack of an effective vaccine against HCV, prevention of disease and eradication have become a huge challenge.

HCV is a member of the *Hepacivirus* genus in the *Flaviviridae* family. It is an enveloped virus with a 9.6kb plus-sense single-stranded RNA genome. It is hepatotropic and gains entry into the hepatocytes via sequential binding to its putative receptors and clathrin-mediated endocytosis, followed by fusion of the viral envelope with endosomal membrane, resulting in the release of the viral genome into the cytoplasm (3). The viral genome is translated into a single polypeptide of approximately 3,000 amino acids, which is processed by host and viral proteases to yield three structural (Core, E1, and E2) and seven nonstructural (p7, NS2, NS3, NS4A, NS4B, NS5A, and NS5B) proteins (4). HCV relies on host lipids and lipoproteins for multiple aspects of its life cycle (5). Viral genome replication occurs on endoplasmic reticulum (ER)-derived modified membranous compartments termed the “membranous web” closely juxtaposed to the cytosolic lipid droplets that serve as platforms for the assembly of viral nucleocapsids (4, 6). The interconnectivity and close proximity of the HCV core protein-loaded lipid droplets to the HCV replication sites and ER membranes decorated with HCV envelope proteins is presumed to facilitate the formation of the nascent virion by the budding of the nucleocapsid into the ER lumen, resulting in its envelopment (7, 8). Infectious HCV particles are called lipoviroparticles due to their low buoyant density (<1.03 to 1.10 g/mL) and their association with lipoproteins (9, 10). The cell-culture-derived HCV virus (HCVcc) is also associated with apolipoproteins and shares biophysical properties with the virus derived from patients (11). However, the patient-derived HCV particles are more buoyant and display higher specific infectivity in the buoyant fractions (10, 12). The association with apolipoproteins happens in the lumen of the ER (9, 13); however, recent evidence also hints at the association of HCV particles with lipids in a dynamic manner in the extracellular milieu (13).

HCV egress is presumed to occur via the conventional ER-Golgi secretory pathway, which is well corroborated by the fact that HCV coopts the very-low-density lipoprotein (VLDL) secretory pathway for its egress (14, 15). Although research in the past decade has unraveled the mechanisms underlying viral entry, replication, and assembly, our understanding concerning HCV release has been a subject of debate with multiple recent studies supporting both conventional (ER-Golgi) and noncanonical routes of HCV release (15–17). It is important to address how the assembled virus particle traverses through the endomembrane secretory compartment to better understand the mechanism of HCV release.

Transport of newly synthesized proteins from the ER to the Golgi complex is a highly selective and vesicular process. These vesicles are generated by the coat protein complex II (COPII) machinery that directly interacts with the cargo and promotes the bending of ER membrane into vesicular carriers (18). The biogenesis of COPII vesicles and budding happens at ER exit sites (ERES), which collectively represent sites of transitional ER, enriched in SEC16 and COPII coat proteins (19). SEC16 orchestrates the formation of COPII-coated vesicles by providing a platform for the interaction of inner (SEC23 and SEC24) and outer COPII coat proteins (SEC13 to SEC31) (19). We investigated the significance of the early secretory components on the HCV life cycle and attempted to decipher the specific role of the major components of this compartment in the HCV life cycle. Our observations suggest that the early secretory pathway is not only essential for HCV release but also crucial for HCV virion assembly and viral replication.

## RESULTS

### HCV derails global protein secretion.

**FIG 1:**
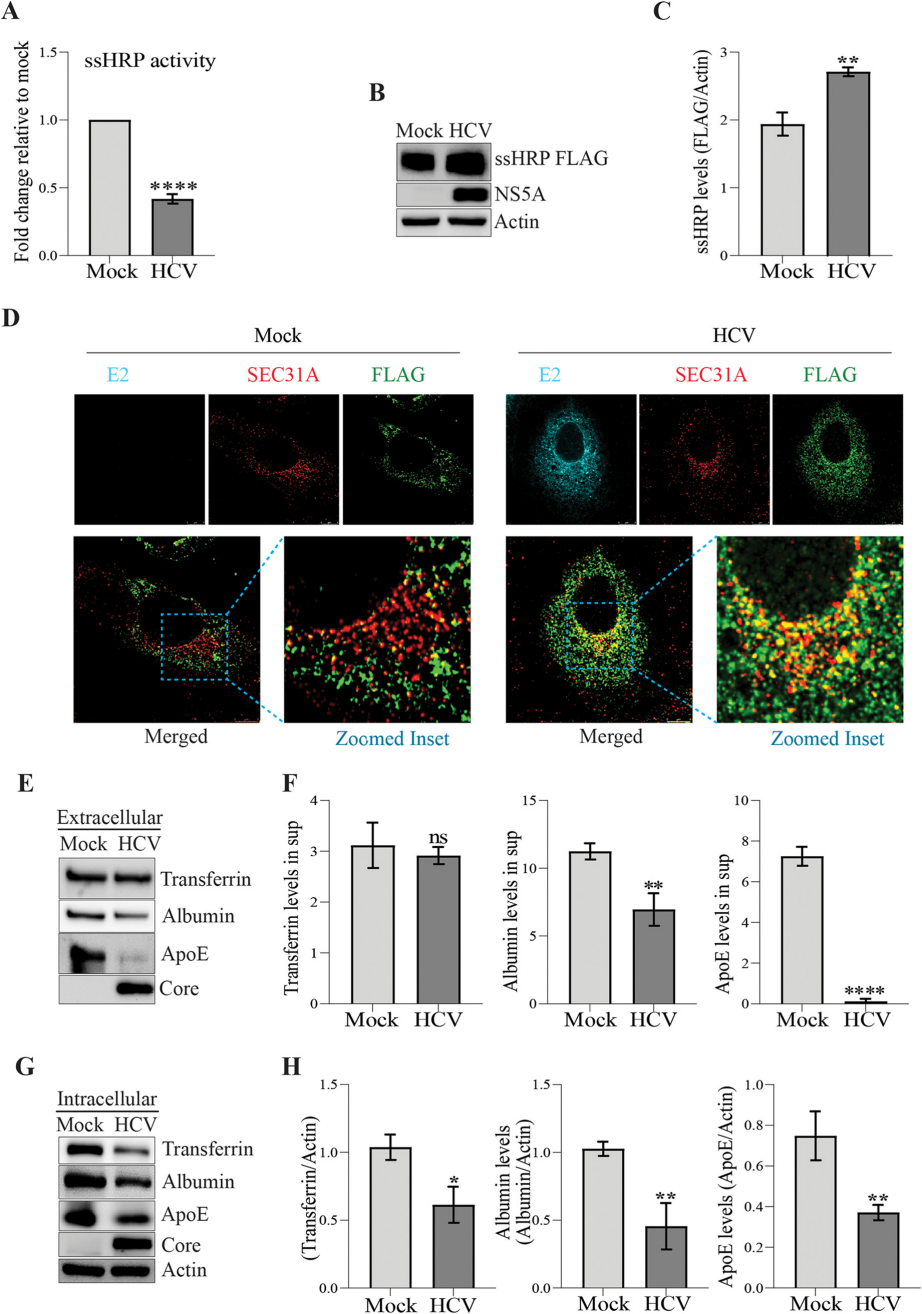
HCV inhibits cellular protein secretion. (A) Relative ssHRP activity in supernatants obtained from mock- or HCV-infected cells. (B) Western blot analysis of ssHRP-FLAG expression in the mock- and HCV-infected Huh7 cells 48 h posttransfection and at 72 hpi. NS5A was used as an infection marker, and β-actin was used as an internal loading control. (C) Densitometry analysis of Western blots shown in panel B. (D) Confocal images representing colocalization between SEC31A used as ERES marker (red) and FLAG (green) in mock- and HCV-infected cells. Envelope (E2) (cyan) was used as an infection marker. Scale bar, 8 μm. (E to H) Western blot and respective densitometry analyses of transferrin, albumin, and ApoE in culture supernatants (E and F) and in cell lysates (G and H) from mock- or HCV-infected cells at 72 hpi. The data are means ± the SEM of three independent experiments. Statistical analysis was done using a Student *t* test (ns, nonsignificant; *, *P* < 0.05; **, *P* < 0.01; ****, *P* < 0.0001).

The delivery of cellular proteins synthesized in the ER to their specific destinations is orchestrated by a complex pathway of intracellular vesicular trafficking (20). Many viruses have been implicated in exploiting the intracellular secretory compartment and reorganizing the cellular membranes to establish viral replication factories and promote viral dissemination (21). How HCV exploits the conventional protein secretory pathway has not been well characterized. To characterize the effect of HCV on global protein secretion, we utilized the well-established HCV cell culture model which replicates the entire HCV life cycle using the HCV genotype 2a chimera Jc1 infection in the human hepatoma cell line, Huh7. Infection of Huh7 cells at a multiplicity of infection (MOI) of 3 with HCV Jc1 virus resulted in a time-dependent increase in viral gene expression and spread of infection (see Fig. S1A to C in the supplemental material). We observed nearly 70 to 90% infected cells between 48 and 72 h postinfection (hpi); hence, we used this MOI throughout this study. Utilizing the ssHRP-FLAG reporter, which expresses a secretory form of peroxidase (HRP containing N-terminal signal sequence) released into the culture media through the conventional ER-Golgi secretory route (22, 23), we characterized the effect of HCV infection on conventional protein secretion. Analysis of secreted HRP activity in the culture supernatants obtained from mock- and HCV-infected cells suggests that HCV downregulates the cellular protein secretion (Fig. 1A). In correlation, we observed higher levels of ssHRP-FLAG in HCV-infected cells in comparison to cells mock infected at 72 hpi (Fig. 1B and C), which indicates a defect in the secretion of ssHRP-FLAG had led to its accumulation within the cells. Confocal images of ssHRP-Flag-transfected mock- and HCV-infected cells immunostained for SEC31A, a COPII outer coat protein commonly enriched in the ER exit sites (ERES) also confirmed the retention of ssHRP-Flag protein at the ERES in HCV-infected cells in comparison to mock-infected cells (Fig. 1D). We did not observe any difference in the transfection efficiency between mock- and HCV-infected cells, as adjudged by the reporter activity of the cotransfected *Renilla* luciferase reporter at 48 h posttransfection (see Fig. S1D). We performed Western blot analysis of liver-specific secretory proteins (24, 25) in mock- and HCV-infected cell lysates and their respective culture supernatants. To avoid contamination from fetal bovine serum present in the cell culture media, the cells were thoroughly washed with phosphate-buffered saline (PBS) after 60 hpi and incubated for a further 12 h in serum-free culture medium, which was subsequently used to determine the levels of secreted proteins. The observations suggest that HCV does not affect transferrin secretion but reduces the secretion of albumin and ApoE (Fig. 1E and F). However, we also observed low intracellular levels of these proteins in HCV-infected cells (Fig. 1G and H). Hence, we examined whether HCV promotes their degradation through proteasome and/or autophagy. Inhibition of autophagy by bafilomycin A1 (a vacuolar H^+^-ATPase inhibitor that prevents lysosomal degradation) (26) resulted in the accumulation ApoE, transferrin, and albumin, suggesting that these proteins undergo autophagic degradation in both mock- and HCV-infected cells (see Fig. S1E). However, the level of degradation through autophagy was more pronounced in HCV-infected cells, as evidenced by the higher accumulation of proteins upon bafilomycin A1 treatment (see Fig. S1F). In contrast, treatment with MG132 a 26S proteasome inhibitor (27) did not lead to the significant accumulation of ApoE, transferrin, and albumin, suggesting that proteasomal degradation is not involved (see Fig. S1E and F in the supplemental material). Interestingly, despite enhanced degradation of transferrin in HCV-infected cells through autophagy, the extracellular levels were similar to that in mock-infected cells, which may be due to the higher release of transferrin from HCV-infected cells (Fig. 1E and F). Alternatively, low transferrin receptor levels and reduced transferrin binding and iron uptake in HCV-infected cells (28) may lead to the accumulation of transferrin in the culture supernatants. Overall, these observations suggest that general protein secretion is perturbed in HCV-infected cells.

### HCV reorganizes the ERES and ERGIC.

**FIG 2:**
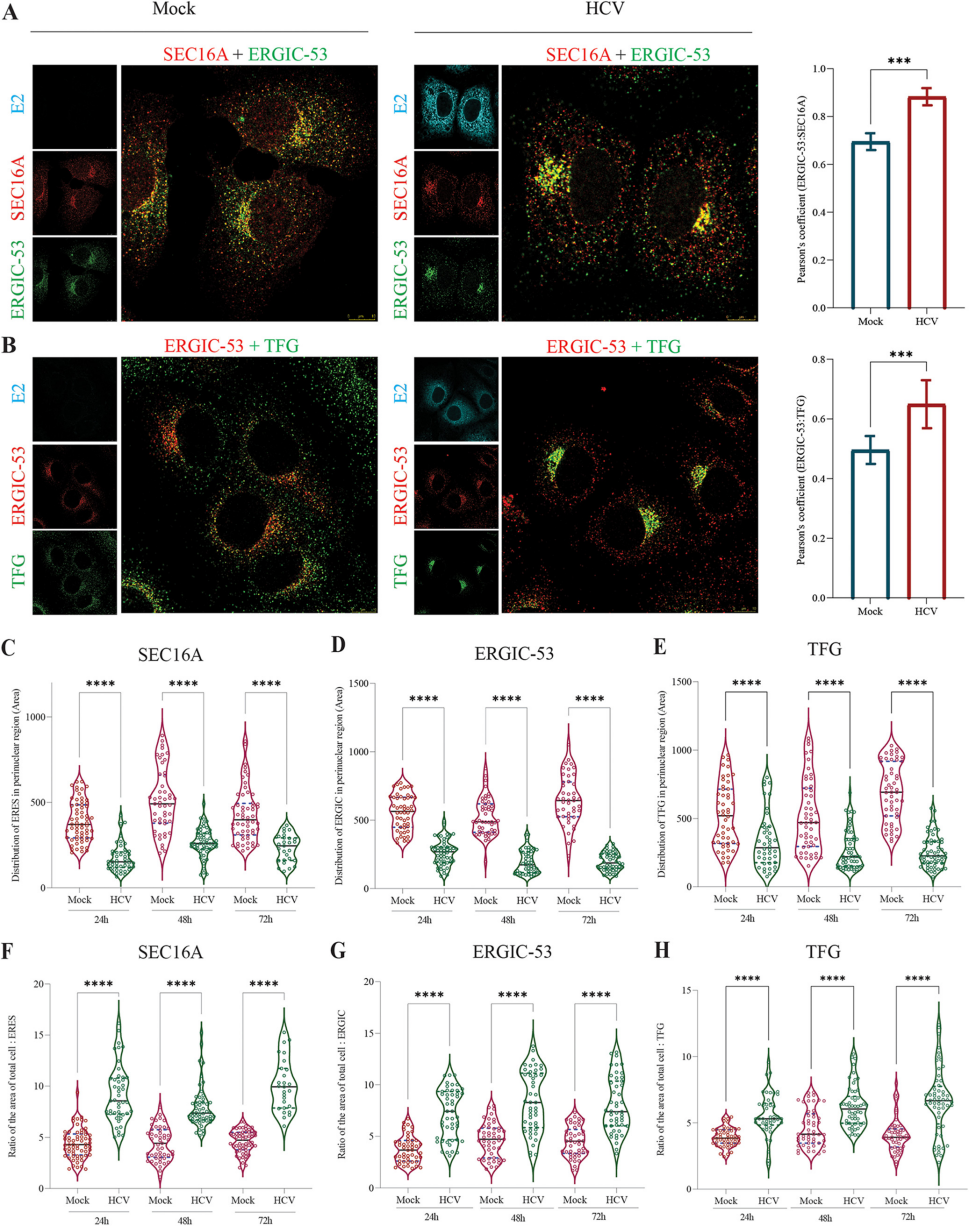
HCV-triggered morphological changes in the early secretory components. (A and B) Confocal microscopy images of Huh7 cells infected with HCV at 72 hpi. (A) Cells were immunostained with SEC16A (ERES marker, red), ERGIC-53 (ERGIC marker, green), and HCV E2 (infection marker, cyan). (B) Cells were immunostained with ERGIC-53 (red), TFG (green), and HCV E2 (infection marker, cyan). The bar graph on the right indicates Pearson’s colocalization coefficient between SEC16A-ERGIC-53 and ERGIC-53-TFG, respectively. (C to H) Image analysis of SEC16A, ERGIC-53, and TFG for perinuclear distribution (C to E) and the total cellular area/area ratio covered by ERES/ERGIC/TFG (F to H). Scale bar, 10 μm. The data presented are means ± the SEM of three independent experiments. Statistical analysis was done using one-way ANOVA (***, *P* < 0.001; ****, *P* < 0.0001).

The discrete sites on the ER membrane involved in COPII vesicle biogenesis are collectively termed ERES (29). SEC16A is an ERES-localized protein that governs the formation of COPII-coated structures by serving as a scaffold for the assembly of the COP-II vesicle via interaction with inner and outer COPII coat proteins (30). The COPII vesicles carry the cargo from the ER to ER-Golgi intermediate compartments (ERGIC) or the Golgi compartment. The ERGIC is believed to act as an intermediate sorting and processing compartment between the ER and Golgi compartment (29). Due to the observed defect in global protein secretion in HCV-infected cells, we further examined whether HCV affects the organization of the ERES and ERGIC. Confocal microscopy analysis of ERES and ERGIC in HCV-infected cells at 24, 48, and 72 hpi, using SEC16A and ERGIC-53 as ERES and ERGIC markers, respectively, showed an overall decline in the expression of SEC16A and ERGIC-53 (Fig. 2A); however, we observed a slight increase in the colocalization between SEC16A and ERGIC-53, as adjudged by the Pearson’s colocalization coefficient (Fig. 2A). Subsequent image analysis suggested that HCV infection resulted in an overall decline in perinuclear distribution of these proteins (Fig. 2C and D), and the infected cells mostly display compact ERES and ERGIC strictly localized to the perinucleus, as determined by the high cell/ERES or cell/ERGIC area ratios in the infected cells (Fig. 2F and G). In contrast, the mock-infected cells display both perinuclear and peripheral distributions of SEC16A and ERGIC-53 with intense perinuclear staining as expected (Fig. 2A), which was also confirmed by image analysis (Fig. 2F to G).

Trk-fused gene (TFG) is an integral component of ERES and plays a vital role in the organization of ERES into large higher-order structures to support the secretion of large cargos such as procollagen (29). TFG is known to interact with SEC16 and the COPII inner coat protein, SEC23 (31, 32), and downregulation of TFG expression is associated with reduced protein secretion from the ER (33). We also investigated the expression and subcellular localization status of TFG in HCV-infected cells. As with SEC16A and ERGIC-53, we also observed that TFG subcellular localization is affected in HCV-infected cells. We observe that both mock- and HCV-infected cells display TFG enrichment in the perinuclear region; however, in HCV-infected cells, the distribution is more compact and is only restricted to the ERES in comparison to the mock-infected cells, which display TFG localization both at ERES and other cellular regions (Fig. 2B; see also Fig. S2A). In comparison to mock-infected cells, TFG colocalization with ERGIC-53 increased in HCV-infected cells, as adjudged by the Pearson colocalization coefficient (Fig. 2B). Similar to what we observed with SEC16A and ERGIC-53, we observed a decline in the overall perinuclear distribution (Fig. 2E) and relative compactness in HCV-infected cells (Fig. 2H). Overall, our analysis suggests that HCV promotes the compactness of ERES and ERGIC, as adjudged by the strict localization of SEC16A, TFG, and ERGIC-53 into smaller compact structures in the perinuclear region in comparison to mock-infected cells, which display bigger and diffused ERES and ERGIC in the perinuclear region (Fig. 2C and H).

**FIG 3:**
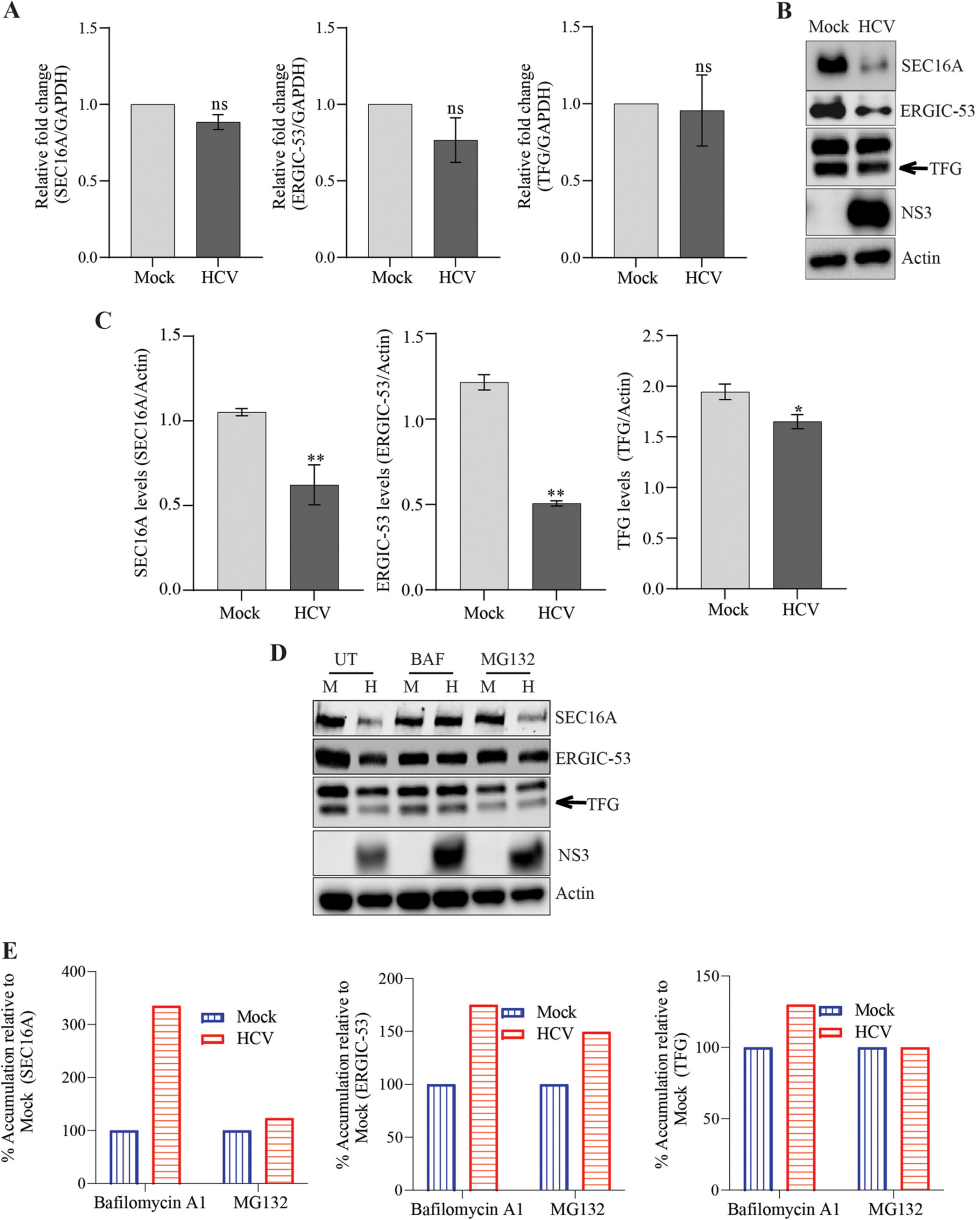
Expression status of ERES- and ERGIC-resident proteins during HCV infection. (A) Bar graph depicting relative transcript levels of SEC16A, ERGIC-53, and TFG in mock- and HCV-infected cells at 72 hpi. (B) Western blot analysis of SEC16A, ERGIC-53, and TFG in mock- and HCV-infected cells at 72 hpi. NS3 is used as an infection marker and β-actin as an internal loading control. (C) Densitometry analysis of the respective Western blots shown in panel B. (D and E) Western blot analysis (D) and respective densitometry of SEC16A, ERGIC-53, and TFG (E) in the mock- or HCV-infected cells at 72 hpi either untreated or subjected to treatment with bafilomycin A1 for 8 h to inhibit autophagosome-lysosome fusion or with MG132 for 4 h to inhibit proteasomal degradation before collection of the cells. NS3 was used as an infection marker and β-actin was used as an internal loading control. In panel E, bar graphs depict the relative percent accumulation of the respective proteins upon treatment with bafilomycin A1 or MG132. Accumulation in mock-treated cells was considered 100%. The data are means ± the SEM of three independent experiments. Statistical analysis was done using a Student *t* test (ns, nonsignificant; *, *P* < 0.05; **, *P* < 0.01).

We further validated the transcript levels and expression status of SEC16A, ERGIC-53, and TFG in HCV-infected cells at 72 hpi (Fig. 3A to C). Although we did not observe any significant change in the transcript levels of SEC16A, ERGIC-53, and TFG (Fig. 3A), we did observe significant decreases in the protein levels of SEC16A, ERGIC-53, and TFG in HCV-infected cells (Fig. 3B and C), which is in agreement to our confocal image analysis (Fig. 2A and B). Next, we determined whether this reduction is associated with autophagy or proteasomal degradation. The mock- and HCV-infected cells were treated with bafilomycin A1 or MG132 to inhibit autophagy and proteasomal degradation. Inhibition of autophagy facilitated the accumulation of SEC16A, ERGIC-53, and TFG (Fig. 3D and E), suggesting that they are degraded through autophagy or are consumed in the autophagy process (34). We also observed that proteasomal inhibition leads to a slight accumulation in ERGIC-53 levels, suggesting that a minor fraction of ERGIC-53 is also subjected to proteasomal degradation during HCV infection (Fig. 3D and E). Overall, these observations suggest that HCV alters the organization of ERES and ERGIC from normal to compact morphology during infection. This may lead to disruption in conventional protein secretion through the ER-Golgi secretory pathway. A similar phenotype is observed in nutrient stress-induced autophagy, which is known to promote ERES aggregation and inhibit ER-Golgi transport (35).

### ERES and ERGIC support multiple aspects of the HCV life cycle.

To determine the significance of the components of the early secretory pathway in the HCV life cycle, we knocked down the proteins that have been implicated to play a critical role in the function of these compartments, using a pool of gene-specific small interfering RNAs (siRNAs). SEC16A is an important regulator of ERES homeostasis, whereas TFG is known to play a role in the transport of large cargo by organizing higher-order ERES (30, 33, 36). ERGIC-53 is a nonglycosylated hexameric type I integral membrane protein that regulates the trafficking of soluble glycoproteins through the ERGIC (37). To determine the effect of knockdown of these proteins on virus entry, replication, and secretion, we adopted two strategies involving siRNA-mediated knockdown before infection (to determine the role in entry and replication) or postinfection (to determine the role in assembly and release). To confirm that the knockdown of SEC16A, TFG, and ERGIC-53 does not affect cell viability, we performed an lactate dehydrogenase (LDH) release assay, which suggested that transfection with respective siRNAs did not lead to a loss in cell viability (see Fig. S2B).

**FIG 4:**
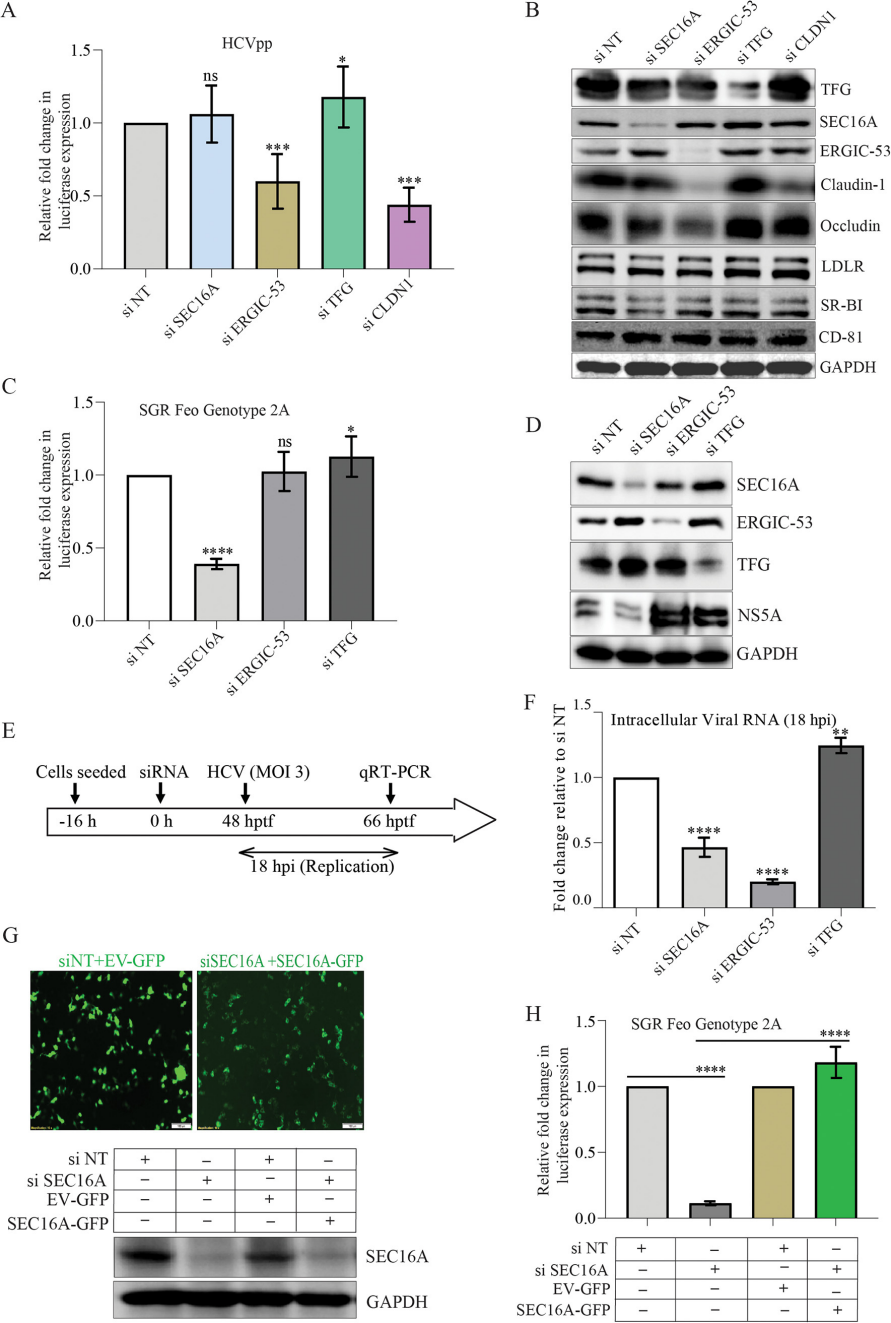
Role of ERES and ERGIC in HCV entry and replication. (A) Bar graph depicting the relative fold change in luciferase reporter activity in SEC16A, ERGIC-53, TFG, and Claudin-1 (CLDN-1) knockdown cells transduced with HCV pseudotyped particles (HCVpp). (B) Western blot analysis depicting the expression status of cell surface receptors in Huh-7 cells knockdown for SEC16A, ERGIC-53, TFG, and Claudin-1. GAPDH was used as an internal loading control. (C) Bar graphs representing the relative fold change in luciferase reporter activity in HCV genotype 2a subgenomic replicon (SGR-Feo) cells transfected with siNT, siSEC16A, siERGIC-53, and siTFG at 48 h posttransfection. (D) Western blot analysis depicting the knockdown efficiency from the subgenomic replicon cells transfected with respective siRNA. (E) Illustration representing the experimental timeline and approach used to determine the role of ERES, ERGIC, and TFG in HCV replication in the HCV cell culture model. (F) Bar graphs representing the fold change relative to siNT in HCV genome copies at 18 hpi. (G) Immunofluorescence images and Western blot analysis depicting the overexpression of SEC16A-GFP or EV-GFP and the SEC16A knockdown efficiency. (H) Bar graph depicting the relative fold change in luciferase reporter activity in SGR-Feo cells transfected with either siNT, siNT plus empty vector (EV-GFP), siSEC16A, or siSEC16A plus SEC16A-GFP. The respective overexpression vectors were transfected at 24 h posttransfection with the respective siRNAs. The luciferase reporter activity was determined 72 h after siRNA transfection. Scale bar, 100 μm. The data presented are means ± the SEM of three independent experiments. Statistical analysis was done using a Student *t* test (ns, nonsignificant; *, *P* < 0.05; **, *P* < 0.01; ****, *P* < 0.0001).

To determine the role of these proteins in HCV entry, we first transfected the cells with respective siRNAs and, at 48 h posttransfection, the cells were infected with HCV-pseudotyped virus particles (HCVpp) harboring firefly luciferase reporter (Fig. 4A). On the basis of the firefly luciferase activity, we observed that knockdown of ERGIC-53 leads to a significant inhibition in HCVpp entry in comparison to si non-targeting (siNT) control (Fig. 4A). As a positive control we knocked down the HCV receptor claudin-1, which also resulted in the inhibition of HCVpp entry (Fig. 4A). ERGIC-53 plays a vital role in glycoprotein trafficking and many of the cell surface glycoproteins like glycosaminoglycans have been implicated to assist HCV adhesion to the cell membrane (3, 38). To characterize how ERGIC-53 knockdown is leading to inhibition in HCV entry, we determined the expression status of some well-known HCV receptors. Western blot analysis suggested that the knockdown of ERGIC-53 resulted in a significant reduction in the levels of claudin-1 and occludin (Fig. 4B), the tight-junction proteins known to play a crucial role in HCV entry (39). We did not observe any change in the levels of other HCV receptors, such as low-density lipoprotein receptor (LDLR), SRB1, and CD81 (Fig. 4B). Confocal imaging of E-cadherin, a key component of the adherens junctions (40), suggested that transient knockdown of the early secretory pathway component did not lead to any major adverse effect on the membrane homeostasis (see Fig. S2C).

To determine the role of the early secretory pathway in HCV replication, we silenced the expression of SEC16A, TFG, and ERGIC-53 in Huh7.5 cells stably harboring the HCV genotype 2a subgenomic replicon (Fig. 4C). Estimation of firefly luciferase activity at 48 h post-siRNA transfection suggested that SEC16A knockdown resulted in the inhibition of HCV replication (Fig. 4C). However, the knockdown of TFG and ERGIC-53 did not lead to any reduction in luciferase activity, suggesting that they are not involved in HCV replication (Fig. 4C). Western blot analysis of HCV NS5A expression in the HCV subgenomic replicon cells transfected with respective siRNAs also confirmed reduced viral gene expression in cells with SEC16A knockdown (Fig. 4D). To further validate our observation in HCV subgenomic replicon cells, we performed a similar experiment using HCV Jc1 virus. We initially transfected the Huh7 cells with gene-specific siRNAs and 48h posttransfection the cells were infected at an MOI of 3 with HCV Jc1 virus and, at 18 hpi, the HCV genome replication efficiency was determined by estimating the intracellular HCV genome copies (Fig. 4E). In agreement with our observation for HCV subgenomic replicon cells, we observed that SEC16A knockdown led to a significant reduction in HCV replication in comparison to siNT controls (Fig. 4F). TFG knockdown did not lead to any reduction in HCV replication, but ERGIC-53 knockdown resulted in a significant decline in HCV replication (Fig. 4F). However, our observations with HCVpp and subgenomic replicon indicated that ERGIC-53 is required for HCV entry but not essential for HCV replication, suggesting that the reduced viral genome copies observed in ERGIC-53 knockdown cells infected with HCV is a consequence of inhibition in HCV entry (Fig. 4A, C, and F). TFG is shown to play a role in antiviral signaling (41); therefore, we assume that the slightly higher levels of luciferase activity (Fig. 4C) or HCV genome copies (Fig. 4F) observed in TFG knockdown condition may be a consequence of reduced antiviral signaling.

Overexpression of SEC16A-green fluorescent protein (GFP) in the subgenomic replicon cells subjected to SEC16A knockdown was able to rescue HCV replication, as indicated by the increase in luciferase activity in comparison to subgenomic replicon cells transfected with the corresponding empty vector (Fig. 4G to H). SEC16A plays a crucial role in intracellular trafficking and is also required for autophagy (42). Both of these cellular processes are crucial for the establishment of HCV replication complex by governing the transport of crucial host factors and/or by providing membrane source for replication platforms via autophagy (43). This may be the cause of negative effect of SEC16A knockdown on HCV replication (Fig. 4C, F, and H).

Many reports suggest that HCV relies on the autophagy pathway for its replication (44). ERES and ERGIC have been implicated to play a crucial role in the initiation of autophagy by serving as a platform for the assembly and regulation of ATG proteins and/or by providing the membrane precursors for phagosome biogenesis (34, 45–47). To determine whether the negative effect of the knockdown of SEC16A on HCV replication is due to a defect in autophagy in these cells, we assessed the autophagy flux using the traffic light reporter ptf-LC3-mRFP-EGFP (see Fig. S3A) in SEC16A, ERGIC-53, and TFG knockdown conditions (48). In line with previous reports, treatment with EBSS to induce autophagy in the respective knockdown cells resulted in slightly lower levels of global autophagy flux in comparison with siNT control (see Fig. S3A and B). Inhibition of lysosomal degradation by treatment with bafilomycin A1 resulted in the low accumulation of the lipidated form of LC3B (LC3BII) in SEC16A and ERGIC53 knockdown in comparison to siNT control cells (see Fig. S3C). TFG knockdown did not lead to any difference in LC3BII levels but resulted in substantial decline in LC3BI levels in HCV-infected cells. Interestingly, bafilomycin A1 treatment in TFG knockdown HCV-infected cells did not lead to any accumulation in LC3BII levels (see Fig. S3D). ERGIC-53 knockdown resulted in decline in the autophagy flux (see Fig. S3A and B) and a decline in LC3B levels (see Fig. S3C), which is suggestive of a negative effect on autophagy; however, we did not observe any effect of ERGIC-53 knockdown on HCV replication in the subgenomic replicon cells (Fig. 4C).

**FIG 5:**
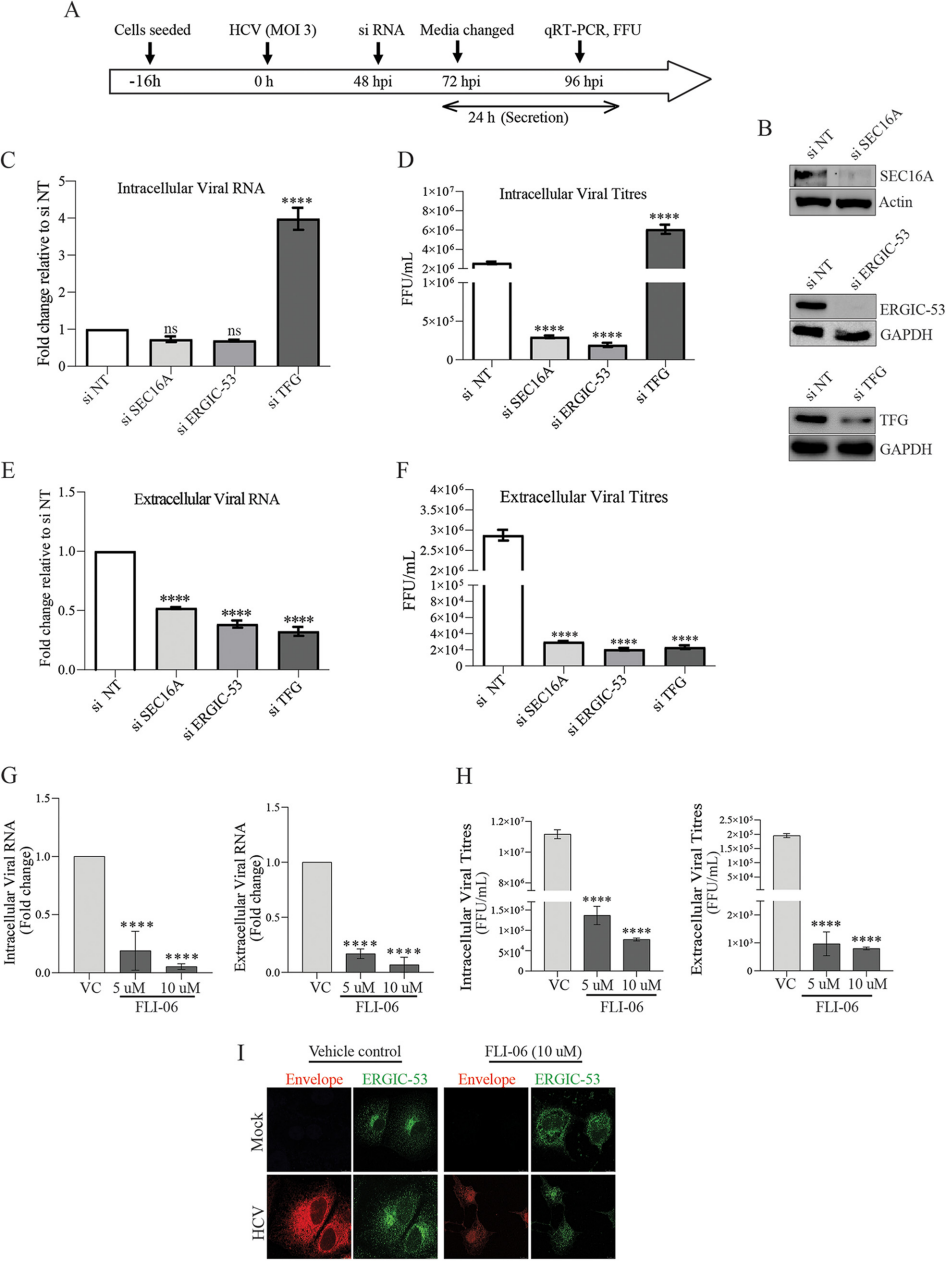
ERES and ERGIC role in HCV assembly and secretion. (A) Illustration representing the experimental timeline and approach used to determine the role of ERES, ERGIC, and TFG in HCV assembly and secretion. (B) Western blot analysis depicting the knockdown efficiency of SEC16A, ERGIC-53, and TFG in cells transfected with respective siRNA. β-Actin and GAPDH were used as internal loading controls. (C to F) Bar graphs representing the relative fold change in the intracellular (C) and extracellular (E) HCV genome copies and the relative change in intracellular (D) and extracellular (F) infectious viral titers in HCV-infected cells at 96 hpi and 48 h posttransfection with siRNAs against SEC16A, ERGIC-53, and TFG. (G and H) Bar graphs representing the relative fold change in the level of intra- and extracellular viral RNA levels (G) and infectious virus titers (H) in HCV-infected cells 48 hpi subjected to 24-h treatment with the indicated concentrations of FLI-06. Dimethyl sulfoxide was used as vehicle control (VC). (I) Confocal images representing the ERGIC (green) morphology and HCV envelope (E2, red) distribution in cells treated with DMSO (VC) or 10 μM FLI-06 for 24 h. Scale bar, 10 μm. The data presented are means ± the SEM of three independent experiments. Statistical analysis was done by using a Student *t* test (ns, nonsignificant; ****, *P* < 0.0001).

**FIG 6:**
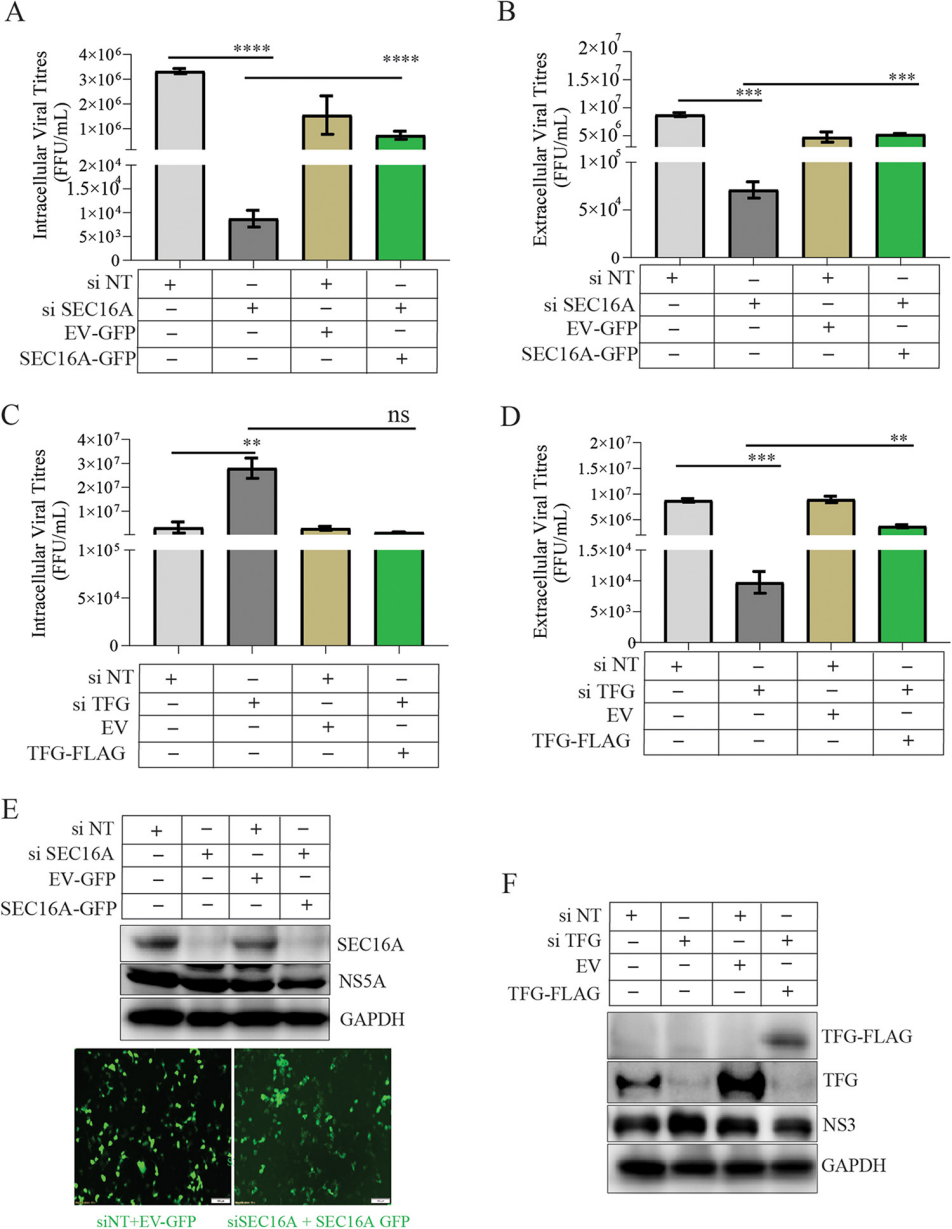
SEC16A and TFG overexpression rescue the HCV assembly and release in SEC16A and TFG knockdown cells. (A to D) HCV-infected Huh7 cells at 48 hpi were transfected with siNT, siSEC16A, siTFG siRNAs and 24 h later transfected with the SEC16A-GFP (A and B) or TFG-FLAG (C and D), respectively. siNT-transfected cells were transfected with corresponding empty vectors (EV-GFP or EV) at 24 h posttransfection with the non-targeting siRNA. HCV infectious titers were determined in the culture supernatants, and cells by using an FFU assay. The bar graphs depict the intra- and extracellular viral titers from the HCV-infected Huh7 cells subjected to respective experimental conditions. (E) Western blot analysis and immunofluorescence images depicting the SEC16A knockdown efficiency and overexpression of SEC16A-GFP or EV-GFP. NS5A and GAPDH were used as an HCV infection marker and an internal loading control, respectively. Scale bar, 100 μm. (F) Western blot analysis depicting the TFG knockdown efficiency and overexpression of TFG-FLAG in HCV-infected Huh7 cells. NS3 and GAPDH were used as an HCV infection marker and an internal loading control, respectively.

To determine the role of these proteins in the later steps of the HCV life cycle (assembly and release of virus particle), we adopted the strategy of silencing the respective genes at 48 hpi and determined the effect of the knockdown on HCV assembly and release at 48 h posttransfection (Fig. 5A). We observed that silencing of SEC16A and ERGIC-53 (Fig. 5B) did not lead to any significant decline in the intracellular viral RNA levels 48 h posttransfection (Fig. 5C), but we did observe a significant decline in intracellular viral titers, extracellular viral RNA, and viral titers (Fig. 5D to F), suggesting that SEC16A and ERGIC-53 are also crucial for viral particle assembly and release. In contrast, silencing TFG expression resulted in higher levels of intracellular viral RNA and viral titers (Fig. 5C and D), whereas, the extracellular viral RNA and viral titers were dramatically low (Fig. 5E and F) suggestive of its specific role in HCV release. To further validate these findings, we treated the HCV-infected cells with FLI-06, an inhibitor of ERES and Golgi transport and a disrupter of Golgi structure (49). Treatment of HCV-infected cells at 48 hpi with nontoxic concentrations of 5 and 10 μM FLI-06 (see Fig. S2D) for 24 h resulted in a significant reduction in both the intra- and extracellular viral RNA and infectious viral titers (Fig. 5G and H), suggesting that the components of the early secretory pathway play a crucial role in multiple aspects of the HCV life cycle. In line with its inhibitory effect on the vesicular protein trafficking between the ER and Golgi, we observed that treatment of HCV-infected cells with 10 μM FLI-06 for 24 h resulted in complete disruption of ERGIC-53 and HCV envelope subcellular organization (Fig. 5I). Overexpression of SEC16A-GFP in HCV-infected cells silenced for SEC16A expression partially rescued the intra- and extracellular viral titers (Fig. 6A and B). Similarly, overexpression of TFG in HCV-infected cells silenced for TFG expression reduced the accumulation in intracellular viral titers (Fig. 6C) and resulted in rescue of HCV release evident by increase in the extracellular virus titers (Fig. 6D). SEC16A-GFP expression was observed in immunofluorescence images (Fig. 6E), but we failed to detect SEC16A-GFP band in the Western blot. TFG-Flag expression was detected by Western blot analysis for Flag (Fig. 6F).

To investigate whether the reduction in intracellular viral titers in SEC16A and ERGIC-53 knockdown is due to the effect of the knockdown on intracellular lipid droplets (LDs), which play a central role in HCV viral particle assembly (6), we performed confocal imaging to determine LD morphology and colocalization with HCV core protein. Confocal imaging revealed that the knockdown of SEC16A dramatically reduced the lipid droplet number and its association with HCV core, whereas ERGIC-53 knockdown cells resulted in slightly large and a smaller number of LDs in comparison to siNT control (see Fig. S3E). In contrast, TFG knockdown cells display moderately higher number of lipid droplets with strong colocalization with HCV core protein in comparison to siNT cells (see Fig. S3E). This may be due to higher levels of viral replication and gene expression observed under TFG knockdown conditions (Fig. 4C and F; see also Fig. 5C and D). Overall, these observations support the negative effect of SEC16A and ERGIC-53 knockdown on HCV virus particle assembly and release.

### Role of COPII coat proteins in HCV life cycle.

**FIG 7:**
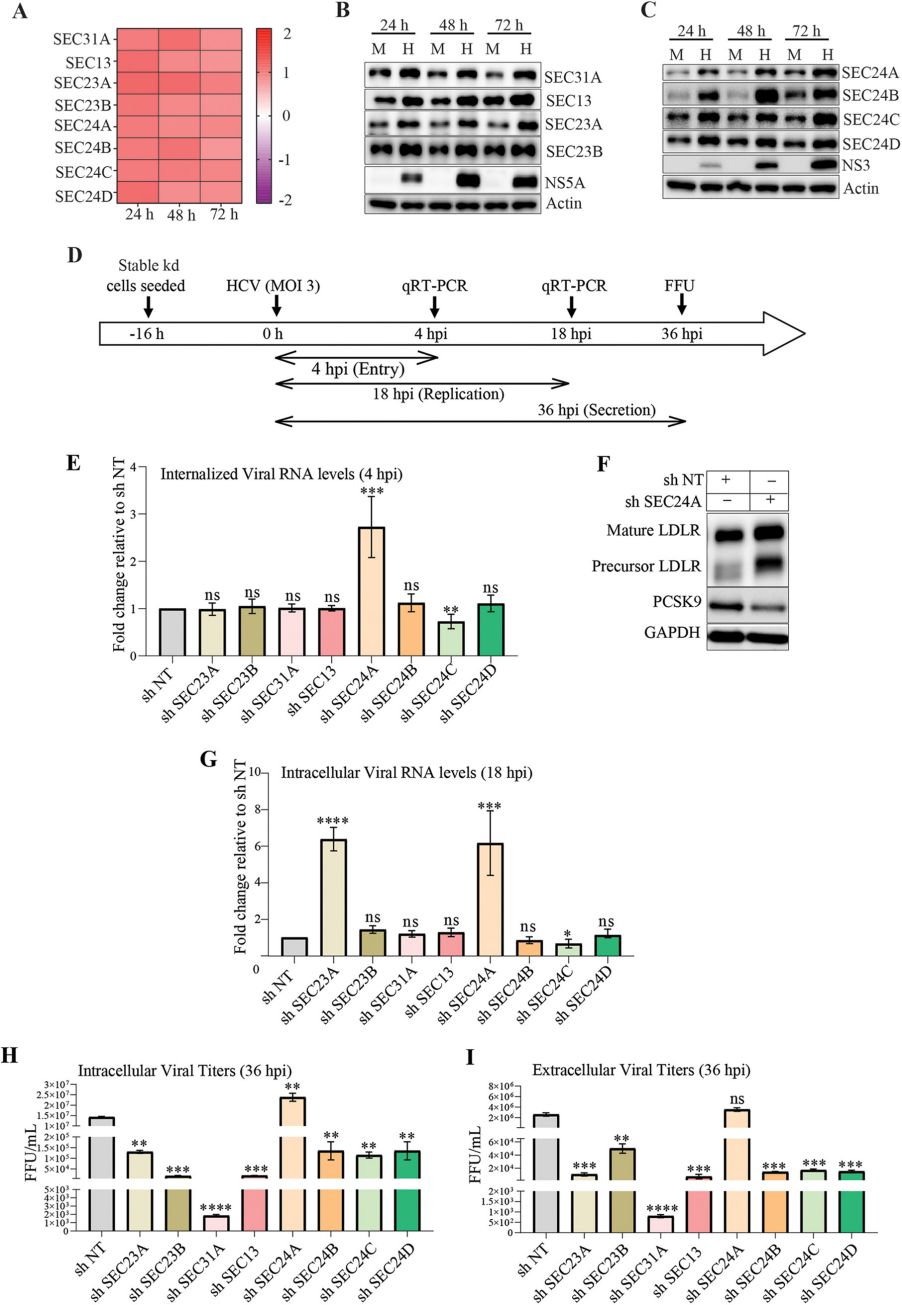
Status of COP-II coat proteins during HCV infection. (A) Heat map representing the transcript level of the COPII outer and inner coat protein genes at the indicated time points postinfection. (B and C) Western blot analysis of the expression of indicated COPII outer (B) and inner (C) coat proteins in mock- or HCV-infected cells at the indicated time points postinfection. HCV NS3 and NS5A were used as infection markers, and β-actin was used as an internal loading control. (D) Illustration representing the experimental timeline and approach used to determine the role of COPII coat protein’s knockdown effect on HCV entry, replication, and secretion. (E) Bar graph representing the fold change in internalized HCV RNA levels at 4 hpi (virus entry). (F) Western blot data representing the LDLR and PCSK9 levels in SEC24A knockdown and shNT control cells. GAPDH is used as an internal loading control. (G to I) Bar graphs representing the relative fold change with respect to the shNT control in the intracellular HCV genome copies at 18 hpi (virus replication) (G) and intracellular virus titers (H) and extracellular virus titers at 36 hpi (I) in the respective COPII coat protein knockdown cells. The data presented are means ± the SEM of three independent experiments. Statistical analysis was done using a Student *t* test (ns, nonsignificant; *, *P* < 0.05; **, *P* < 0.01; ***, *P* < 0.001; ****, *P* < 0.0001).

Onward transport of the protein cargo from the ER to the Golgi is facilitated by the COPII vesicles generated at the ERES (50). The COPII coat proteins promote the capture of cargo and ER membrane curvature to drive the COPII vesicle formation. The SEC23-SEC24 heterodimers and the SEC13-SEC31 heterotetramers (two units of each subunit) constitute the inner coat and outer coat proteins, respectively (50). To identify the specific role of individual COPII coat proteins, we assessed their significance in different aspects of the HCV life cycle. Initially, we determined their expression status during infection by both qPCR and Western blot analysis. We observed a substantial increase in the levels of the COPII coat proteins in HCV-infected cells compared to mock-infected cells; however, we did not observe a correlative increase in the transcript levels (Fig. 7A to C; see also Fig. S4A), suggesting that the increase in proteins levels is not a consequence of enhanced gene expression in HCV-infected cells (Fig. 7A to C). Inhibition of autophagosome-lysosome fusion by treatment with bafilomycin A1 resulted in the accumulation of all the COPII inner and outer coat proteins in both mock- and HCV-infected cells (see Fig. S4B). However, the comparative analysis of band intensities suggested that the magnitude of accumulation was higher in mock-infected cells than in HCV-infected cells (see Fig. S4C). This suggests that COPII coat proteins undergo autophagosomal degradation under basal conditions (51) and indicates that the high levels of COPII coat proteins observed in HCV-infected cells (see Fig. S4B) may be a consequence of inhibition of autophagy flux during HCV infection (44, 52, 53).

We generated stable knockdown cells of various COPII coat proteins using lentiviruses harboring shRNA against the respective genes. The knockdown efficiency of the respective COPII coat proteins in the stable cell lines was determined by Western blot analysis (see Fig. S5A). We observed around 80 to 90% knockdown efficiency in most of the inner coat proteins, whereas we obtained 50 to 70% knockdown efficiency in outer coat proteins (see Fig. S5A). Further, to evaluate the effect of these knockdowns on conventional ER cargo secretion, we performed an ssHRP secretion assay in these stable knockdown cell lines. As expected, the knockdown of the COPII coat proteins resulted in a significant decline in the ssHRP release (see Fig. S5B). Despite high knockdown efficiency of the COPII coat proteins, we did not observe a complete block in the conventional ER-Golgi transport as determined by the ssHRP assay, suggesting that low levels of COPII coat proteins are enough to partially support the COPII vesicle-mediated secretion.

We infected the stable COPII coat protein knockdown cells at an MOI of 3 with HCV and collected the cells and culture supernatants at 4, 18, and 36 hpi to determine the effect of the knockdown on virus entry, replication, assembly, and secretion (Fig. 7D). Determination of the levels of internalized HCV RNA at 4 hpi revealed that knockdown of the outer coat (SEC31A and SEC13) and the majority of the inner coat proteins (SEC23A, SEC23B, SEC24B, and SEC24D) did not affect virus entry (Fig. 7E). However, the knockdown of the coat protein SEC24A resulted in enhanced entry and the knockdown of SEC24C resulted in slight inhibition of viral entry (Fig. 7E). SEC24C is shown to interact with claudin-1 and promote its transport to the plasma membrane. Claudin-1 is a receptor for HCV entry and a defect in its transport to the plasma membrane negatively affects HCV entry (54). SEC24A has been reported to regulate the expression levels of LDLR (55). SEC24A is required for the transport of proprotein convertase subtilisin/kexin type 9 (PCSK9), which negatively regulates LDLR levels (56). In agreement, we also observed high levels of LDLR and low levels of PCSK9 expression in SEC24A stable knockdown cells (Fig. 7F). LDLR serves as a receptor for HCV entry (57), which correlates with our observation of enhanced HCV entry in SEC24A knockdown cells (Fig. 7E).

To determine the effect of the knockdown of COPII coat proteins on HCV replication, we quantified the intracellular HCV RNA levels in the respective cells at 18 hpi (Fig. 7G). Our results indicated that the knockdown of inner coat proteins SEC23A and SEC24A resulted in higher intracellular viral RNA copies in comparison to nontargeted shRNA control cells (Fig. 7G), whereas the knockdown of inner coat proteins SEC23B, SEC24B, SEC24C, and SEC24D and outer coat proteins SEC31A and SEC13 did not show any significant change in the intracellular viral RNA levels (Fig. 7G). Increase in HCV genome copies in SEC24A knockdown cells may be a consequence of enhanced viral entry. However, the high levels of HCV genome copies in SEC23A knockdown may be a consequence of enhanced viral replication. In correlation to reduced viral entry, we also observe slightly reduced intracellular HCV genome copies in SEC24C knockdown (Fig. 7G).

Determination of the intra- and extracellular HCV viral titers at 36 hpi suggests that the COPII coat proteins knockdown affects viral particle assembly and release (Fig. 7H and I). We also noticed that the inhibitory effect of the COPII coat proteins knockdown is more profound on viral particle release than on the assembly (compare the intracellular versus extracellular virus titers) (Fig. 7H and I). This suggests that the COPII coat proteins are not only involved in viral assembly but also contribute to viral release, thus resulting in a higher percentage reduction in the extracellular viral titers in the knockdown conditions. Interestingly, the knockdown of the outer coat proteins resulted in higher percent reduction in the extracellular virus titers compared to the knockdown of the inner coat proteins (Fig. 7H and I). In correlation to enhanced viral entry and replication observed in SEC24A knockdown cells, we also observe high levels of intracellular viral titers (Fig. 7H). However, the magnitude of the difference between the sh non-targeting (shNT) control and shSEC24A is reduced in the intracellular viral titers, suggesting that the knockdown is having a negative effect on viral assembly (Fig. 7H). In addition, the extracellular viral titers in SEC24A knockdown cells were similar to that observed in the shNT control (Fig. 7I), suggestive of inhibition in viral release and an overall reduction in extracellular viral titers due to defect of ER-Golgi transport in SEC24A knockdown, similar to that observed with knockdown of other COPII coat proteins (see Fig. S5B). Overall, these results suggests that knockdown of COPII inner and outer coat proteins negatively affects the HCV assembly and secretion.

## DISCUSSION

The conventional cellular protein secretion pathway involves the sequential transport of cargo from the ER to different cellular destinations or extracellular milieu through the Golgi by vesicle transport (58). Noncanonical secretion involves the secretion of leaderless proteins through pores in the plasma membrane or membrane-bound organelles or secretion of proteins with signal peptide to the plasma membrane via routes that bypass the Golgi complex (59). Noncanonical protein secretion is induced by cellular stresses, which may compromise the functional integrity of the classical secretory pathway (60). Many viruses have been shown to exploit the secretory pathway and the endomembrane system to promote viral processes such as genome replication, viral particle assembly, and egress (21). Viral infections are usually associated with physiological stresses such as ER stress and oxidative stress (61), which may induce unconventional protein secretion.

In this study, we observe that HCV disrupts the normal organization of the ERES and ERGIC compartments of the endomembrane system and inhibits cellular protein secretion (Fig. 2 and 1D). HCV promotes the compactness of these compartments during infection (Fig. 2). A similar phenotype is observed in turnip mosaic virus-infected cells, which display a perinuclear globular structure due to the amalgamation of ER, Golgi, and COPII coat proteins, resulting in the inhibition of protein secretion (62). Subversion of COPII vesicles for the formation of viral replication sites has been reported during tombusvirus (63) and poliovirus infections. Polio virus is known to increase the pool of membrane precursors for establishing a replication complex by enhancing the biogenesis of COPII vesicles in the early phase of infection (64).

It is believed that HCV traverses the conventional ER-Golgi secretory route for its egress (14, 15, 65). However, there is a lack of strong evidence and many conflicting reports suggest alternate routes of HCV egress. In line with the conventional secretory pathway, the knockdown of SAR1A, a small GTPase protein that initiates the biogenesis of COPII-coated vesicles, or knockdown of Rab1b a small GTPase which promotes the fusion of COPII vesicles with Golgi bodies, negatively affects HCV virus secretion. Similarly, a number of Golgi resident proteins involved in conventional secretion, such as the phosphatidylinositol-4-kinase β, clathrin interactor 1 (CLINT1), and GOLPH3 a phosphatidylinositol-4-phosphate (PI4P) interacting protein that tethers Golgi bodies with the cytoskeleton, have been implicated in HCV secretion (16, 65). Many reports also implicate noncanonical routes of HCV release and demonstrate the involvement of late endosomes/multivesicular bodies (66) and ESCRT machinery (67). The lack of Endo H-sensitive glycosylation pattern in HCV glycoproteins further supports the absence of HCV glycoprotein processing in the Golgi apparatus (68). A study with a panel of dominant negative Rab GTPases involved in the *trans*-Golgi network to endosome trafficking shows the importance of this pathway in HCV egress (69), suggesting that the cross talk at the post-Golgi pathway is also of significance in HCV release. In agreement, clathrin and AP-1 have been shown to be involved in HCV release either from the *trans*-Golgi network and/or recycling endosomes to the plasma membrane (70).

In this study, we investigated the roles of the early secretory pathway proteins and COPII coat proteins in the HCV life cycle. Our data support the importance of the early secretory components of the ER-Golgi secretory route in HCV replication, assembly, and release (Fig. 4, 5, and 6). Knockdown of crucial ERES protein SEC16A resulted in inhibition of HCV replication (Fig. 4C), whereas the knockdown of ERGIC-53, a crucial component of the ERGIC, specifically inhibited HCV entry (Fig. 4A). ERGIC-53 is a mannose-specific membrane protein that functions as a receptor for glycoproteins and facilitates their trafficking through the ERGIC. Mutation in ERGIC-53 affects glycoprotein trafficking and is associated with coagulation disorders (37). Interestingly, ERGIC-53 is reported to bind to the glycoproteins of many viruses and is assumed to play an essential role in promoting virus attachment to host cells. Interestingly, it has been shown that some viruses lacking ERGIC-53 are fusion incompetent and noninfectious (38). Our experiments with transient knockdown of the SEC16A and ERGIC-53 in HCV-infected cells further established the role of these early secretory components in HCV assembly and release as well (Fig. 5A to F and Fig. 6).

Surprisingly, the knockdown of TFG, another essential ERES component required for organizing the transition of large cargo such as procollagen through the ERES to the Golgi compartment (36), specifically effected HCV release and resulted in the accumulation of intracellular virus particles (Fig. 4 and 5). HCV lipoviroparticles are likely above 100 nm in size and represent atypical ER cargo; hence, TFG may be crucial for HCV transport from the ER to the Golgi compartment (5, 11, 14). In line with this notion, we also observe that HCV promotes TFG translocation to the ERES during infection (Fig. 2B). This suggests that different components of the ERES facilitates distinct aspects of HCV life cycle. TFG is exclusively involved in HCV release, whereas SEC16 is required for HCV replication, assembly, and release, which may be due to the distinct functional aspects of ERES governed by these two proteins.

The biogenesis of COPII vesicles at the ERES involves the interaction of the putative cargo proteins with the inner coat proteins and their incorporation into the budding vesicles. The SEC24 protein, a principal subunit of the inner coat complex, plays a critical role in cargo selection and incorporation into the budding vesicles. As expected, the knockdown of the majority of COPII coat proteins did not perturb viral entry and replication (Fig. 7E and G). Surprisingly, SEC24A knockdown specifically promoted HCV entry (Fig. 7E) due to its role in regulating LDLR on the hepatic cells due to a defect in PCSK9 trafficking to the plasma membrane (55). Although we anticipated that the COPII coat proteins are primarily involved in HCV egress, we observed—to our surprise—that the knockdown of COPII coat proteins resulted in the inhibition of both HCV assembly and release (Fig. 7H and I).

The cross talk between the ERES/ERGIC and autophagy and the role of secretory autophagy in the HCV life cycle cannot be ruled out. It is well established that HCV relies on autophagy machinery for its genome replication. A recent study has shown that blocking autophagy at late stages of autophagosome-lysosome fusion promotes HCV release (71). Whether this is a consequence of the accumulation of autophagy vesicles or an enhanced availability of crucial host factor remains to be characterized. Previous reports suggest that HCV perturbs autophagy flux and increases the steady-state levels of autophagosomes by suppressing their fusion with lysosomes via the cleavage of Rab7 adaptor protein RILP (Rab interacting lysosomal protein) (71, 72) or by regulating the expression of Arl8b or the SNARE protein, syntaxin 17 (73, 74). Silencing Arl8b expression has been shown to inhibit HCV release (74). We also observed that inhibition of autophagy by bafilomycin A1 promoted the intracellular levels of ApoE (see Fig. S1E and F), which has been shown to play a crucial role in HCV assembly and release (75). These studies suggest that the accumulation of autophagosomes positively contribute to HCV release. In agreement, our study also emphasizes the importance of ERES and ERGIC in HCV replication and release (Fig. 4 and 5), which may be due to their crucial role as platforms for the assembly of ATG proteins and phagophore formation or as membrane precursors for autophagosome biogenesis (34, 45–47). We observed that autophagy flux was partly inhibited in SEC16A and ERGIC-53 knockdown cells (see Fig. S3C). In agreement, we observed that the knockdown of SEC16A negatively affects HCV replication, which may be an outcome of the defect in autophagy (see Fig. S3A and C). Defects in the early secretory pathway may also perturb the intracellular transport of proteins and lipids that play a crucial role in HCV replication. In addition, our experiments designed to decipher the role of these proteins in the early and later phases of the HCV life cycle clearly demonstrate that functional ERES and ERGIC are not only essential for viral genome replication but also significantly contribute to HCV egress (Fig. 5 and Fig. 6).

Our findings, in conjunction with the previous reports suggest that the block of ER-to-Golgi trafficking leads to the accumulation of intracellular virus (14, 70), clearly depicting that the early secretory pathway and intra-Golgi pathway are involved in HCV release. This also suggests that the association with the noncanonical routes is probably occurring while transiting toward or through the Golgi stage or at the post-Golgi stage. Surprisingly, in many studies, the effect of the knockdown on HCV release does not strongly correlate with the knockdown efficiency, suggesting that minute expression levels of the implicated proteins are sufficient to facilitate HCV egress or HCV egress is orchestrated through distinct secretory routes that run in parallel in the infected hepatocytes. Studies designed to address all these alternative routes of HCV release would provide more definitive answers to this enigmatic aspect of the HCV life cycle.

## MATERIALS AND METHODS

### Cell culture and HCV infection.

Human hepatoma cell line Huh7 and its derived cell line Huh7.5.1 were cultured in a humidified incubator at 37°C under 5% CO_2_ in Dulbecco modified Eagle medium supplemented with 10% fetal bovine serum, 100 U/mL penicillin, and 100 μg/mL streptomycin, and nonessential amino acids (Invitrogen, Carlsbad, CA). Huh7 was kindly provided by Aleem Siddiqui, UCSD, and Huh7.5.1 were obtained from Apath LLC, New York, NY. For HCV infection, the Huh7 cells were infected at 3 MOI with genotype 2a chimeric virus Jc1 (kindly provided by Ralf Bartenschlager, University of Heidelberg) for all experiments in this study (76).

### HCVcc production and propagation.

The plasmid pFK-J6/C3 harboring HCV (Jc1) was linearized by digestion with XbaI and subjected to *in vitro* transcription with a RiboMAX large-scale RNA production system-T7 (Promega, Madison, WI). First, 10 μg of isolated HCV RNA was used for electroporation of 4 × 10^6^ Huh7.5.1 cells by employing a Bio-Rad Gene Pulser X cell electroporated as described previously (77). The culture supernatant was collected at day 5 postelectroporation, and virus titers were determined by the focus-forming unit (FFU) assay, as described below (78). For propagation of the cell-culture-derived HCV (HCVcc) virus, Huh7.5.1 cells were infected with the virus at an MOI of 0.1, and culture supernatants were collected at days 3, 5, and 7 postinfection. The culture supernatants were clarified by centrifugation, aliquoted in 500-μL fractions, and stored in a −80°C deep freezer until further use. The infectious virus titers in the culture supernatants were quantified using an FFU assay as described below.

### HCV pseudovirus production and transduction to the target cells.

Murine leukemia virus (MLV)-based HCV-pseudotyped particles (79, 80) were generated by cotransfection of a pcDNA JFH E1E2 plasmid expressing MLV Gag-Pol and transfer vector expressing firefly luciferase reporter (kindly provided by Aleem Siddiqui, UCSD) into 293FT cells using a Cal-Phos mammalian transfection kit (TaKaRa, Japan) according to the manufacturer’s instructions. At 48 h posttransfection, supernatants containing HCV pseudovirus were collected, filtered through a 0.45-μm-pore size syringe filter, and transduced into Huh7 cells. The luciferase activity was measured at 48 h postransduction using a Promega Firefly luciferase assay kit in a Thermo Scientific Varioskan Flash multimode reader.

### Estimation of HCV genome copies and infectious titers.

Viral genome copies were quantified by absolute quantification using a PrimeScript one-step RT-PCR kit (TaKaRa, Japan) according to the manufacturer’s instructions with HCV-specific primers and probes set (81) in a Quant Studio 6 Real-Time PCR machine. HCV infectious titers were determined by FFU assay as described previously (14, 15). Briefly, log fold dilutions of the infectious cell culture medium/clarified cell lysates were used to infect naive Huh7.5.1 cells seeded in 96-well culture plates. At 72 hpi, the cells were fixed in 4% paraformaldehyde and immunostained with antibodies against HCV E2 (82). Immunofluorescence imaging was performed in an Olympus DX58 fluorescence microscope, the numbers of HCV-positive foci were counted, and infectious titers were calculated as FFU/mL of culture supernatant.

### RNA extraction and analysis of gene expression.

Total cellular RNA was extracted by TRIzol (Invitrogen, USA) method according to the manufacturer’s protocol. The concentration and quality of isolated RNA were assessed by using a NanoDrop spectrophotometer. Isolated RNA was subjected to reverse transcription and cDNA synthesis with random hexamer primers by using a TaKaRa PrimeScript first-strand cDNA synthesis kit according to the manufacturer’s instructions. Utilizing the gene-specific primers listed in Table S2 in the supplemental material, we performed gene expression analysis with PowerUp SYBR Green Master Mix (Thermo Scientific, Ltd., USA) in a Quant Studio 6 Real-Time PCR machine.

### Immunofluorescence.

Mock- or HCV-infected cells grown on glass coverslips for a stipulated time were fixed in 4% paraformaldehyde, washed three times in PBS, and then permeabilized and blocked for 1 h with 3% bovine serum albumin in PBS with 0.1% Triton X-100. The cells were then probed with the respective primary antibodies overnight at 4°C, followed by PBS washes and subsequent incubation for 1 h at room temperature with Alexa Fluor-conjugated secondary antibodies against the respective primary antibodies. After three washes in PBS, the coverslips were mounted onto Prolong Gold Antifade (Invitrogen, USA). For lipid droplet staining, the fixed cells were stained with the neutral lipid dye BODIPY 493/503 (Invitrogen) for 15 min at room temperature and subsequently mounted onto Prolong Gold Antifade. Images were visualized with a Leica SP8 confocal microscope. Image analysis and quantification were done using ImageJ software.

### Image analysis.

Image analysis was done using ImageJ software. The ImageJ macro Coloc-2 was used to determine colocalization efficiency. Region of interest-based analysis was used to obtain the total cellular area, the perinuclear area, and the ERGIC/ERES-enriched area in a respective cell. Approximately 50 cells were analyzed for each quantification. Total cell fluorescence was calculated by using the following formula: corrected total cell fluorescence = integrated density − (area of selected cell × mean fluorescence of background reading).

### Western blot analysis.

After the respective treatment mock- or HCV-infected cells were washed twice with PBS and lysed on ice in radioimmunoprecipitation assay buffer supplemented with protease and phosphatase inhibitors, followed by brief sonication and centrifugation at 13,000 rpm for 20 min at 4°C to obtain the clarified supernatants. Protein estimation was done using a Pierce BCA protein assay kit (Thermo Scientific, USA) according to the manufacturer’s instructions. An equal amount of protein was taken, and samples were prepared in SDS-PAGE loading dye by heating for 5 min at 95°C. Proteins separated on SDS-PAGE were transferred to nitrocellulose membrane and subjected to Western blot analysis. The nitrocellulose membranes were blocked for 1 h at room temperature in buffer containing 3% bovine serum albumin in PBS with 0.1% Tween 20, followed by overnight incubation with respective primary antibody solution. After 3× PBS + Tween 20 (PBST) washes for 15 min each, the membrane was incubated with corresponding horseradish peroxidase (HRP)-conjugated secondary antibody (Promega, USA) for 1 h at room temperature, followed by 3× PBST washes for 15 min each. The blots were developed using Clarity Western ECL substrate (Bio-Rad, USA) in a Bio-Rad Chemi-Doc MP imaging system. All antibodies used in this study are listed in Table S1.

For Western blot analysis of proteins secreted into the culture medium, cells at 60 hpi were washed twice in PBS, supplemented with serum-free culture media, and then further incubated for 12 h in order to avoid possible contamination with secretory proteins (albumin and transferrin) from the serum. The collected culture medium was concentrated 5-fold using 3-kDa Amicon centrifugal filters. Subsequently, 50-μL portions of the clarified culture supernatants were subjected to Western blot analysis to determine the levels of secreted albumin, transferrin, ApoE, and HCV core protein.

### Generation of transient knockdowns.

To determine the effect of the knockdown on the early events (entry and genome replication) and late events (assembly and release) of the HCV life cycle, we adopted two strategies involving siRNA-mediated silencing of *SEC16A*, *ERGIC-53*, and *TFG* either prior to infection or at 48 hpi.

### (i) siRNA transfection followed by HCV infection.

Huh7 cells were seeded at 70% confluency and subjected to siRNA transfection using Lipofectamine RNAi Max (Invitrogen, USA) according to the manufacturer’s instructions. At 48 h posttransfection, the cells were infected at an MOI of 3 with HCV Jc1 virus. Cells and supernatants were collected at 4, 18, and 36 hpi to determine the knockdown effects on virus entry, replication, assembly, and release, respectively.

### (ii) HCV infection followed by siRNA transfection.

Huh7 cells were infected at an MOI of 3 with HCV Jc1. At 48 hpi, the cells were transfected with respective siRNAs as described above. At 48 h posttransfection, the cells and supernatants were collected to determine the effect of knockdown on viral particle assembly and release.

### Generation of stable knockdown cells.

Lentivirus particles harboring the respective shRNA were produced in 293FT cells by cotransfection of lentiviral shRNA vector (pLKO.1 puro), pCMVR8.74 (Addgene 22036), and pMD2.G (Addgene 12259) with a Cal-Phos mammalian transfection kit according to the manufacturer’s instructions. At 48 h posttransfection, the lentiviral supernatants were collected and passed through 0.45-μm-pore-size filters. The Huh7 cells were transduced with the lentiviral particles in the presence of 8 μg/mL of Polybrene, and at 4 h postransduction, the cells were supplemented with fresh culture media. At 48 h postransduction, transduced cells were selected with 5 μg/mL puromycin for 1 week, and the stable cells were then maintained in 2 μg/mL puromycin (83). The stable knockdown cells were infected at an MOI of 3 with HCV Jc1 virus as per the experimental requirement.

### Cloning of human TFG FLAG.

We cloned the human TFG gene into the mammalian expression vector pCMV 3TAG3A, which has 3-Flag tags at the C-terminal end. Briefly, total RNA was isolated from Huh7 cells using the TRIzol method. The cDNA was then prepared using a PrimeScript cDNA synthesis kit, followed by PCR amplification with TFG-specific primers (see Table S1) harboring the restriction sites. We performed the restriction digestion using HindIII and XhoI and ligated the PCR-amplified TFG cDNA by using T4 DNA ligase to the digested vector overnight at 16°C. The ligated vector was transformed into DH5α Escherichia coli cells. Plasmid DNA was isolated by using a Qiagen plasmid miniprep kit, followed by Sanger sequence analysis to rule out any mutations (41).

### Rescue experiments.

To determine whether the overexpression of SEC16A or TFG can rescue from SEC16A or TFG knockdown phenotype and to rule out the off-target effects of the gene-specific siRNAs, Huh7 cells were infected with HCV (3 MOI) for 48 h, followed by transfection with the respective siRNA pools, and transfected 24 h later with the overexpression vectors pEGFP-SEC16A (Addgene, 36155) or TFG-FLAG (generated in the study) or corresponding empty vectors. At 48 h posttransfection with overexpression vectors, the cells and culture supernatants were collected to perform the required assays. In case of secretion/release assay, the cells, at 24 h posttransfection with overexpression vectors, were washed three times with PBS and supplemented with fresh media. After 24 h, the cells and culture supernatants were collected to determine the intra- and extracellular infectious virus titers, respectively. To demonstrate that the replication defect observed in knockdown cells can be rescued by ectopic expression of the respective proteins, SGR-Feo cells were transfected with the respective siRNAs and transfected 24 h later with overexpression vectors; the intracellular luciferase activity was then determined after another 48 h.

### Autophagy flux assays.

To study the autophagy flux, we used the ptfLC3B vector (Addgene, 21074). Briefly, Huh7 cells grown on coverslips were transfected with respective siRNA and, at 48 h posttransfection, the cells were transfected with autophagy flux reporter ptfLC3B. To check the induction of autophagy with ptfLC3B, at 24 h posttransfection, the cells were either left untreated or treated with EBSS (Earle’s balanced salt solution) for 2 h to induce nutrient-stress-mediated autophagy. Posttreatment cells were fixed in 4% paraformaldehyde and mounted on Prolong Diamond antifade (Invitrogen). Images were acquired in Leica SP8 confocal microscope, and autophagy flux was quantified based on LC3B green/red puncta (48).

### ssHRP-FLAG secretion assay.

ssHRP-FLAG secretion assay was carried out as described previously (22), briefly mock- and HCV-infected Huh7 cells at 24 hpi were cotransfected with ssHRP-FLAG (kindly provided by Vivek Malhotra, Centre for Genomic Regulation, Barcelona, Spain) and *Renilla* luciferase vector (Promega, USA). *Renilla* luciferase activity was used for normalizing transfection efficiency. At 24 h posttransfection, the culture medium was replaced with fresh phenol red-free complete medium. At 48 h posttransfection, the cells and supernatants were collected. Cell lysates were subjected to Western blotting to determine ssHRP expression, and supernatants were used to determine the secreted ssHRP levels by performing an HRP activity assay.

### Statistical analysis.

All experiments were carried out as three independent biological replicates, and the data are presented as means ± the standard errors of the mean (SEM). All statistical analysis was done using a Student *t* test or one-way analysis of variance (ANOVA) in GraphPad Prism 9 according to the experimental requirement based on single- or multiple-parameter analysis to assess the significance level. A *P* value of <0.05 was regarded as significant.
