# Supplementary material for: The Early Secretory Pathway Is Crucial for Multiple Aspects of the Hepatitis C Virus Life Cycle
Source: J Virol. 2023 Jun 20;97(7):e00180-23. doi: 10.1128/jvi.00180-23 (PMC10373535; doi:10.1128/jvi.00180-23)
Supplement: Supplemental file 1 — Fig. S1 to S5. Download jvi.00180-23-s0001.pdf, PDF file, 1.9 MB [file jvi.00180-23-s0001.pdf]

Figure S1

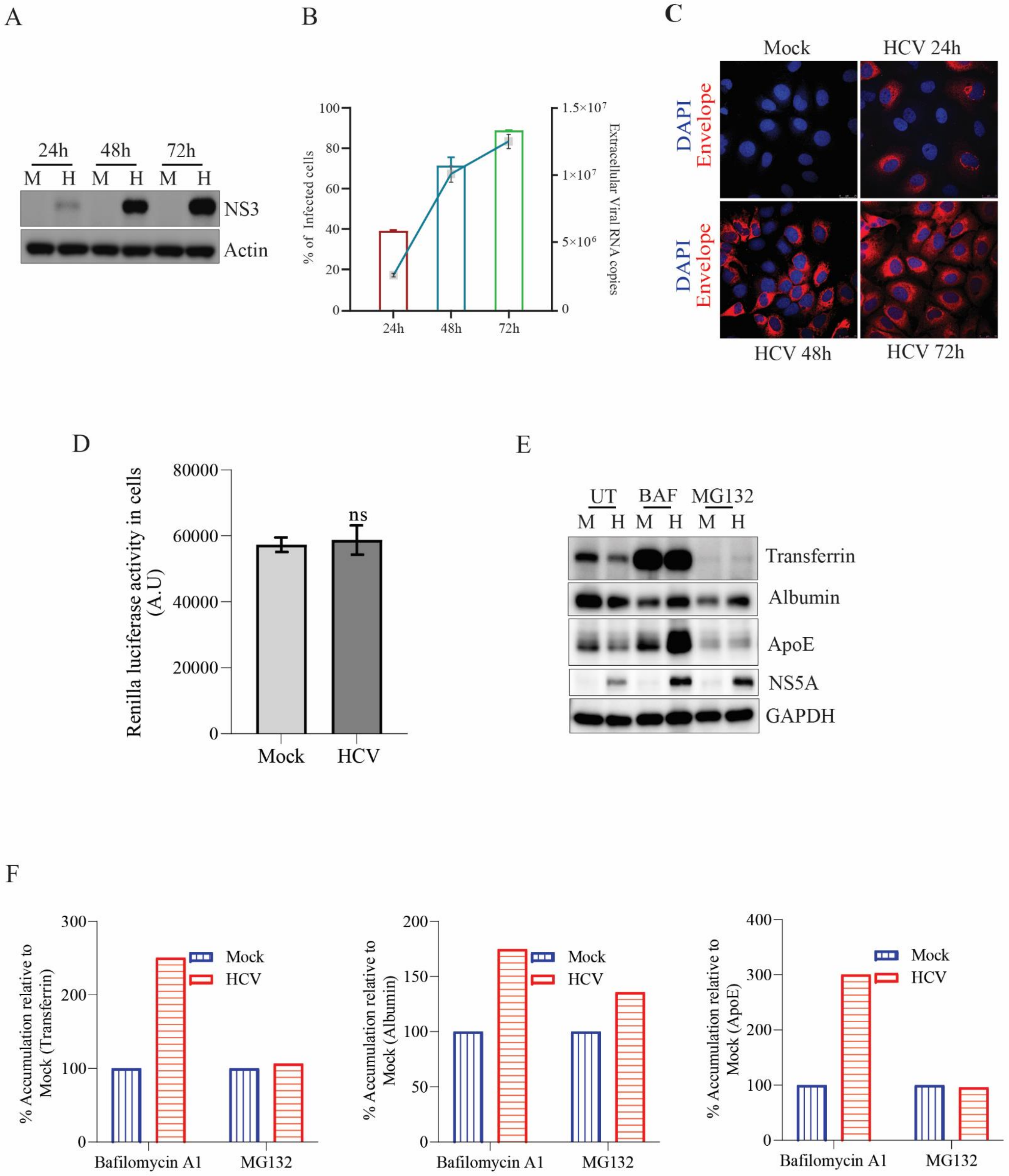

A
24h
48h
72h
M H M H M H
NS3
Actin
B
% of Infected cells
Extracellular Viral RNA copies
100
80
60
40
20
0
1.5×10^7
1×10^7
5×10^6
0
24h
48h
72h
C
Mock
HCV 24h
HCV 48h
HCV 72h
DAPI Envelope
D
Renilla luciferase activity in cells (A.U)
80000
60000
40000
20000
0
ns
Mock
HCV
E
UT
BAF
MG132
M H M H M H
Transferrin
Albumin
ApoE
NS5A
GAPDH
F
% Accumulation relative to Mock (Transferrin)
% Accumulation relative to Mock (Albumin)
% Accumulation relative to Mock (ApoE)
Mock
HCV
Bafilomycin A1
MG132
300
200
100
0
150
50
400

Figure S2

A

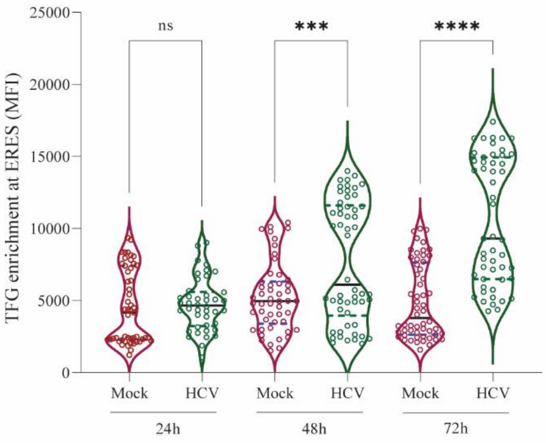

C

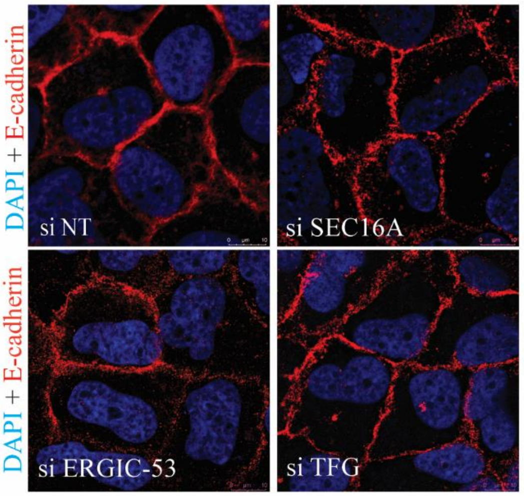

B

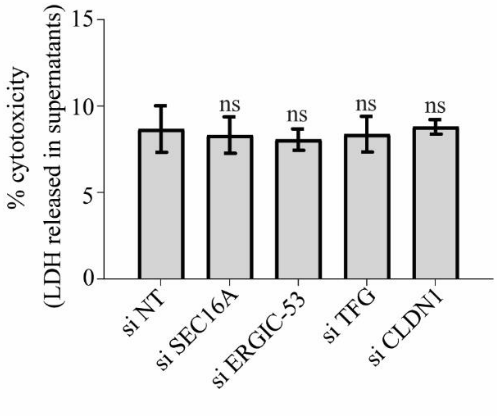

D

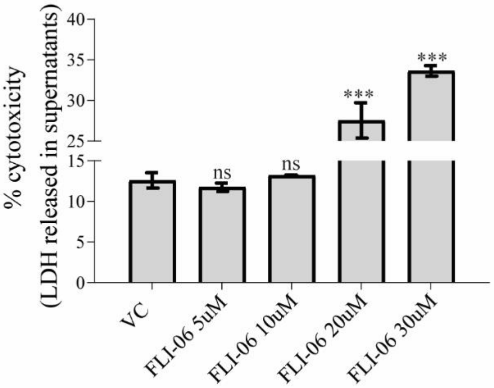

Figure S3

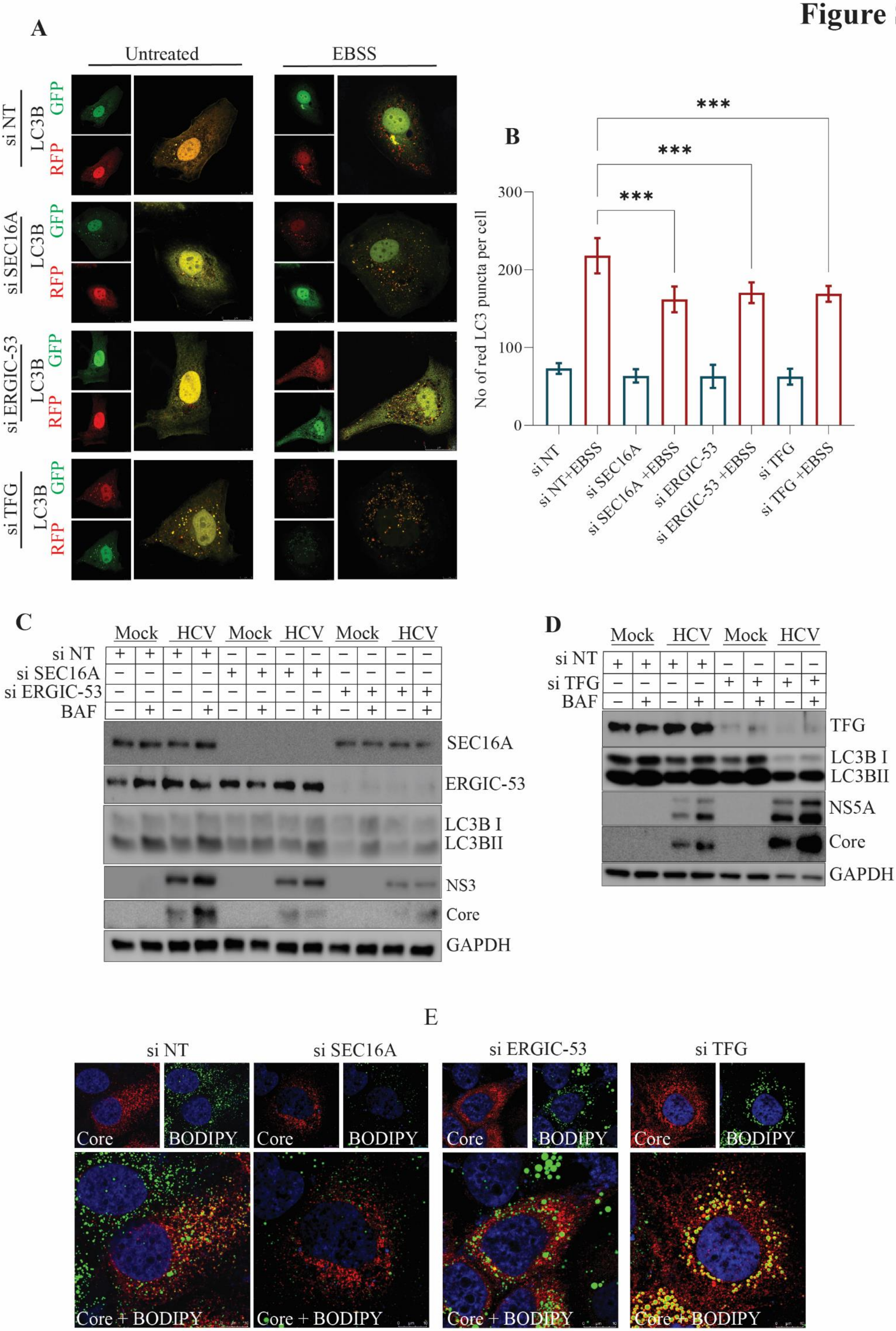
A
Untreated
EBSS
si NT
si SEC16A
si ERGIC-53
si TFG
LC3B
GFP
RFP
B
***
***
***
No of red LC3 puncta per cell
300
200
100
0
si NT
si NT+EBSS
si SEC16A
si SEC16A +EBSS
si ERGIC-53
si ERGIC-53 +EBSS
si TFG
si TFG +EBSS
C
Mock
HCV
si NT
si SEC16A
si ERGIC-53
BAF
SEC16A
ERGIC-53
LC3B I
LC3BII
NS3
Core
GAPDH
D
Mock
HCV
si NT
si TFG
BAF
TFG
LC3B I
LC3BII
NS5A
Core
GAPDH
E
si NT
si SEC16A
si ERGIC-53
si TFG
Core
BODIPY
Core + BODIPY

Figure S4

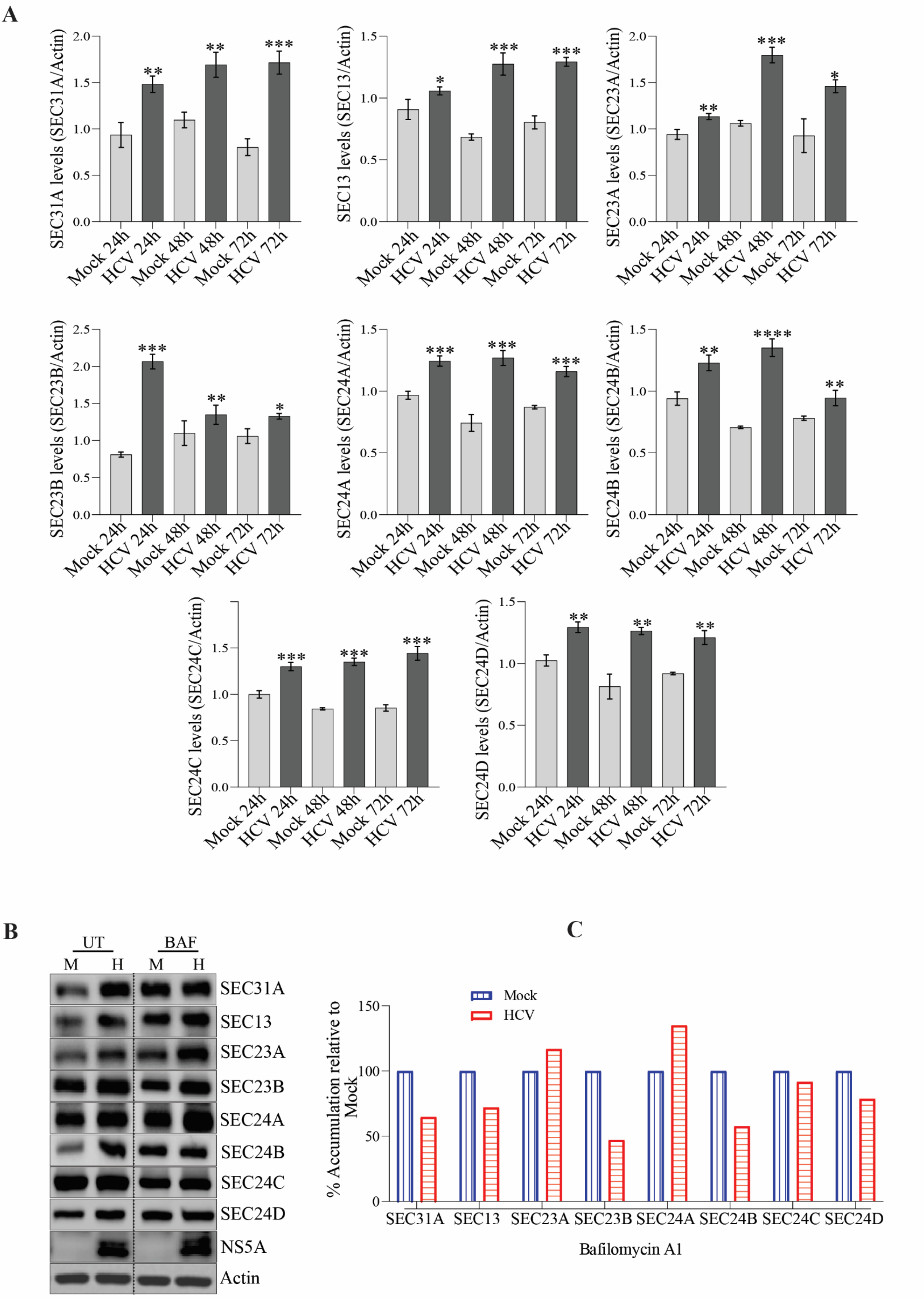
A
SEC31A levels (SEC31A/Actin)
SEC13 levels (SEC13/Actin)
SEC23A levels (SEC23A/Actin)
SEC23B levels (SEC23B/Actin)
SEC24A levels (SEC24A/Actin)
SEC24B levels (SEC24B/Actin)
SEC24C levels (SEC24C/Actin)
SEC24D levels (SEC24D/Actin)
Mock 24h
HCV 24h
Mock 48h
HCV 48h
Mock 72h
HCV 72h
B
UT
BAF
M
H
M
H
SEC31A
SEC13
SEC23A
SEC23B
SEC24A
SEC24B
SEC24C
SEC24D
NS5A
Actin
C
Mock
HCV
% Accumulation relative to Mock
SEC31A SEC13 SEC23A SEC23B SEC24A SEC24B SEC24C SEC24D
Bafilomycin A1

Figure S5

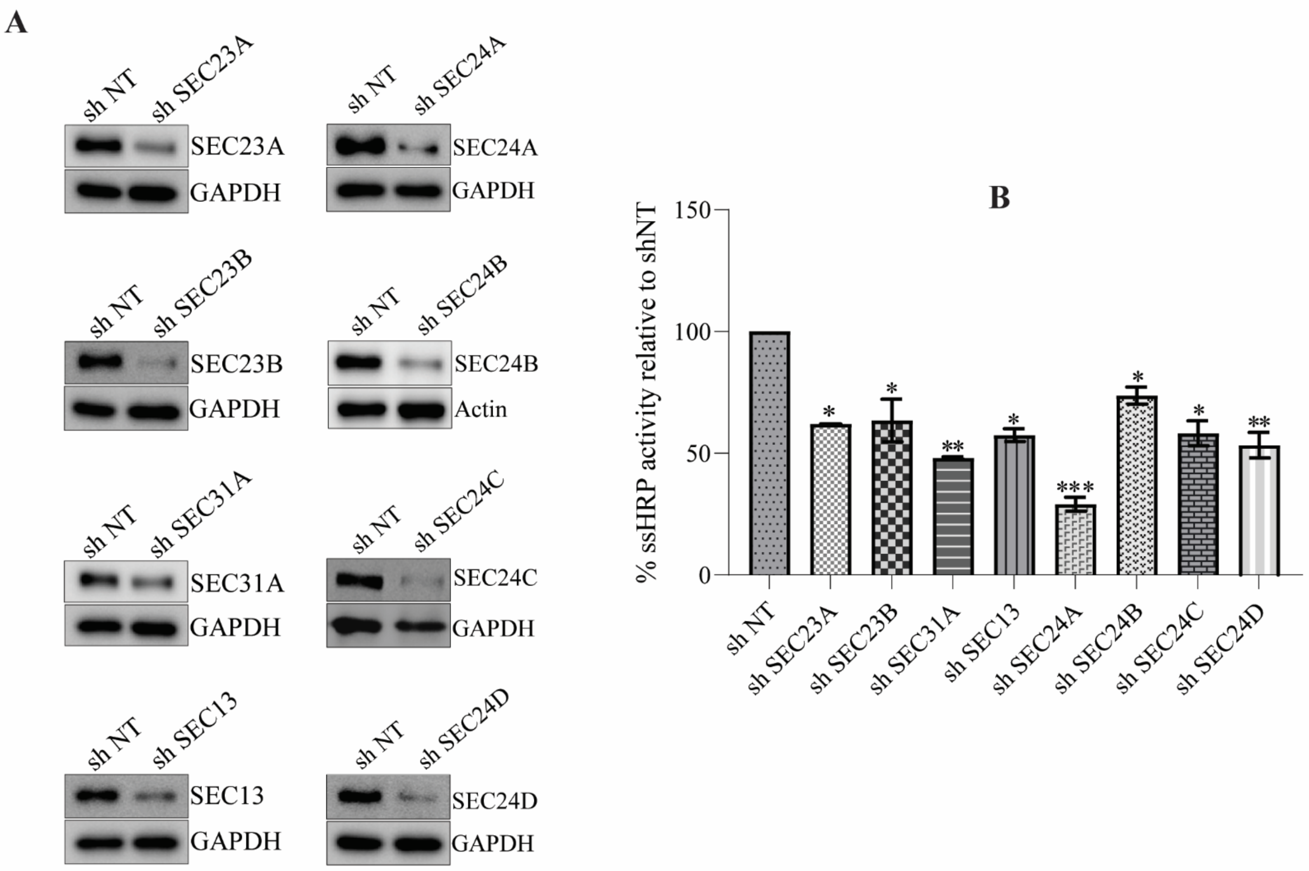
A
sh NT
sh SEC23A
SEC23A
GAPDH
sh NT
sh SEC24A
SEC24A
GAPDH
sh NT
sh SEC23B
SEC23B
GAPDH
sh NT
sh SEC24B
SEC24B
Actin
sh NT
sh SEC31A
SEC31A
GAPDH
sh NT
sh SEC24C
SEC24C
GAPDH
sh NT
sh SEC13
SEC13
GAPDH
sh NT
sh SEC24D
SEC24D
GAPDH
B
% ssHRP activity relative to shNT
150
100
50
0
sh NT
sh SEC23A
sh SEC23B
sh SEC31A
sh SEC13
sh SEC24A
sh SEC24B
sh SEC24C
sh SEC24D
*
*
**
*
***
*
*
**

Supplementary figure legends:

Figure S1: **HCV inhibits global protein secretion.** (A) Western blot analysis of HCV NS3 protein at indicated time points post-infection in mock- or HCV-infected cells. β-actin was used as an internal loading control. (B) Graph representing the percentage of HCV-infected cells and extracellular viral genome copies at the indicated time points post HCV infection. (C) Immunofluorescence images of HCV-infected cells at indicated time points post-infection. HCV E2 was used as an infection marker and nuclei were counterstained with DAPI (scale bar- 25 µm). (D) Relative Renila luciferase activity (co-transfected with ssHRP-FLAG plasmid) in either mock- or HCV-infected cells. (E & F) Western blot analysis (E) and percentage accumulation determined by densitometry analysis (F) of transferrin, albumin, and ApoE from the mock- and HCV-infected cells treated with bafilomycin A1 or MG132 to inhibit degradation via autophagy or proteasome.

Figure S2: (A) Image analysis representing the TFG enrichment at the ERES in the mock or HCV-infected cells. (B) Bar graph depicting the extracellular lactate dehydrogenase (LDH) activity to check for the cytotoxicity in Huh7 cells silenced with respective siRNAs (siNT, siSEC16A, siERGIC-53, siTFG, and siClaudin-1). (C) Confocal images represent the cell membrane homeostasis of E-Cadherin (red) at the plasma membrane in the cells transfected with siSEC16A, siERGIC-53, siTFG, and siNT control, 48h post-transfection (scale bar- 10 µm). Nuclei are counterstained with DAPI (blue).(D) Bar graph depicting extracellular lactate dehydrogenase (LDH) activity in Huh7 cells treated with indicated concentrations of FLI-06 or vehicle control. Statistical analysis was done using student's t-test. ns = nonsignificant; *** = P>; 0.001; **** = P >; 0.0001.

Figure S3: Autophagy flux status in SEC16A, ERGIC-53, and TFG knockdown cells: To study the autophagy flux, SEC16A, ERGIC-53, and TFG silenced cells were transfected with LC3 traffic light reporter plasmid ptf-LC3-EGFP-RFP and either left untreated or subjected to treatment with EBSS to induce nutrient stress-induced autophagy. (A) Representative images of untreated and EBSS-treated cells. (B) Quantification of the number of red LC3 puncta per cell in the knockdown cells. Around 20 cells were analysed for each condition. (C & D) Western blot analysis of LC3B lipidation in mock- or HCV-infected Huh7 cells silenced with respective siRNA (siNT, siSEC16A, siERGIC-53, and siTFG), either untreated or treated with bafilomycin-A1. Antibodies against SEC16A, TFG and ERGIC-53 antibodies were used to determine the knockdown efficiency of respective siRNAs, HCV core, NS3, and NS5A

antibodies were used as infection markers and GAPDH was used as an internal loading control. E) HCV-infected Huh7 cells at 48h post-infection were transfected with respective siRNAs and 48h post-transfection, the cells were fixed and stained for lipid droplets with BODIPY 505 (green), HCV core (red), and nuclei counterstained with DAPI (blue). Confocal images were captured in Leica SP8 confocal microscope. Scale bar =10 µm. The data presented are the mean ± SEM of three independent Experiments. Statistical analysis was done by using the student's t-test. *** = P>; 0.001.

Figure S4: COPII coat protein expression status during the HCV infection (A) Bar graphs depicting the densitometry analysis of the COPII inner and outer coat proteins at indicated time points of either mock or HCV infection. (B) Western blot analysis of COPII coat proteins and percentage accumulation determined by the densitometry analysis, with accumulation in mock considered at 100% (C) from the mock or HCV-infected (72hpi) cells treated with bafilomycinA1. The data presented are the mean ± SEM of three independent experiments. Statistical analysis was done by using the student's t-test. * = P>; 0.05, ** = P>; 0.01; *** =P >; 0.001; **** = P &>; 0.0001.

Figure S5: Western blot data representing the knockdown efficiency of COPII coat proteins. (B) Bar graphs depicting the global protein secretion status determined by the ssHRP assay in the Huh7 cells knockdown for the respective COPII coat proteins. The data presented are the mean ± SEM of three independent experiments. Statistical analysis was done by using the student's t-test. * = P>; 0.05, ** = P>; 0.01; *** =P >; 0.001.
