# Supplementary material for: The Early Secretory Pathway Is Crucial for Multiple Aspects of the Hepatitis C Virus Life Cycle
Source: J Virol. 2023 Jun 20;97(7):e00180-23. doi: 10.1128/jvi.00180-23 (PMC10373535; doi:10.1128/jvi.00180-23)
Supplement: Supplemental file 2 — Tables S1 and S2. Download jvi.00180-23-s0002.pdf, PDF file, 0.2 MB [file jvi.00180-23-s0002.pdf]

	Table S1		
**S/N**	**Name**	**Cat number**	**Identifier**
1	Renilla Luciferase Assay System	E2810	Promega
2	Lipofectamine™ RNAi-MAX Transfection Reagent	13778150	Invitrogen
3			
4	FLI-06	SML0975-5MG	Sigma-Aldrich
5	TMB substrate set	421101	Bio legend
6	Clarity Western ECL Substrate	1705061	Bio-Rad
7	Pierce™ BCA Protein Assay Kit	23225	Thermo Scientific™
8	SYBRTM Green Master Mix.	A25741	Applied Biosystems
	**siRNA**		
1	si GENOME Non-Targeting siRNA Pool #2	D-001206-14-20	Dharmacon
2	si GENOME Human LMAN1 (3998) siRNA – SMART pool.	M-012122-00-0005	Dharmacon
3	si GENOME Human SEC16A (9919) siRNA – SMART pool.	M-026032-01-0005	Dharmacon
4	si GENOME Human TFG siRNA – SMART pool.	M-016366-01-0005	Dharmacon
5	si GENOME Human CLDN1siRNA – SMART pool.	M-017369-01-0005	Dharmacon
	**shRNA**		
1	SEC23A Human shRNA (TRCN0000232513)	SHCLNG-NM_006364	Sigma-Aldrich
2	SEC23B Human shRNA (TRCN0000381493)	SHCLNG-NM_006363	Sigma-Aldrich
3	SEC24A Human shRNA (TRCN0000253655)	SHCLNG-NM_021982	Sigma-Aldrich
4	SEC24B Human shRNA (TRCN0000246106)	SHCLNG-NM_006323	Sigma-Aldrich
5	SEC24C Human shRNA (TRCN0000380070)	SHCLNG-NM_004922	Sigma-Aldrich
6	SEC24D Human shRNA (TRCN0000065170)	SHCLNG-NM_014822	Sigma-Aldrich
7	SEC31A Human shRNA (TRCN0000146890)	SHCLNG-NM_014933	Sigma-Aldrich
8	SEC13 Human shRNA (TRCN0000064985)	SHCLNG-NM_030673	Sigma-Aldrich
	**Antibodies**		
1	Anti -ApoE rabbit monoclonal	CST	#13366
2	Anti-Albumin	SCBT	SC-271605
2	Anti -beta-actin rabbit monoclonal	CST	#4970
3	Anti-ERGIC-53 mouse monoclonal	SCBT	SC-398777
4	Anti-FLAG M2 clone M2	Sigma-Aldrich	F1804
5	Anti-GAPDH rabbit polyclonal	SCBT	SC-25778
6	Anti-GRASP-65	SCBT	SC-374423
7	Anti-LC3B	Sigma-Aldrich	L7543
8	SEC16A Rabbit polyclonal	Thermo Scientific™	PA5-52182
9	SEC23A Rabbit polyclonal	Abcam	Ab-137583
10	SEC23B rabbit monoclonal	Thermo Scientific™	MA5-38169
11	SEC24A Rabbit polyclonal	CST	9678
12	SEC24B rabbit monoclonal	CST	12042

13	SEC24C rabbit monoclonal	CST	14676
14	SEC24D rabbit monoclonal	CST	14687
15	SEC31A mouse monoclonal	SCBT	SC-376587
16	SEC13 Goat polyclonal	SCBT	SC-103196
17	SQSTM/p62	Bio bharati	
18	Transferrin mouse monoclonal	SCBT	Sc-365871
19	Anti-TGN46	Sigma-Aldrich	T7576
20	Anti-HCV NS3mouse monoclonal	Abcam	Ab65407
21	Anti-HCV Core mouse monoclonal	Thermo Scientific™	MA1-080
22	Anti-HCV NS5A rabbit polyclonal	GeneTex	GTX131272
23	HCV E2 anti human antibody	Kind gift from Mansunlaw	
24	Claudin-1 Rabbit monoclonal antibody	Abclonal	A21971
25	Occludin Rabbit polyclonal antibody	Abclonal	A2601
26	CD81 Rabbit monoclonal antibody	Abclonal	A4863
27	E-Cadherin (24E10) Rabbit mAb	CST	#3195
28	SEC31A mouse monoclonal antibody	SCBT	376587

Table S2

S/N	Primers	Sequence
1	SEC13 FP	GGGAAGGCCAATGGGAAGTA
2	SEC13 RP	CGATGGGTGGTCTATGAGGC
3	SEC31A FP	CTCAAGATGGAAGCCACCCT
4	SEC31A RP	TGGCTTGAGTGAGTTGCACA
5	SEC23A FP	GGTGGCACATGTCAGTGGAA
6	SEC23A RP	ACGCCCTCCTTGAGGAATTG
7	SEC23B FP	TCTTCCGAGGGACCAAGGAT
8	SEC23B RP	AAGCAAAAGGGTGCTCCTGT
9	SEC24A FP	GAATTCAGTTTGCCAGAGTTTGT
10	SEC24A RP	CAAATGTTATGAAGCCAATTTTTG
11	SEC24B FP	GAATTCAGTTTGCCAGAGTTTGT
12	SEC24B RP	CAAATGTTATGAAGCCAATTTTTG
13	SEC24C FP	CGTCTCCTACAATGCCATCAGG3
14	SEC24C RP	GGTGACAAAGCCAACGCGGATT
15	SEC24D FP	TGGACCAGTCAGATGCAACAGG
16	SEC24D RP	CCAGTCTTCTTCCAATGTGGTCC
17	SEC16A FP	CACAGACATGAGGCACTGAAA
18	SEC16A RP	CCTGCCATGGAGCAAGTT
18	ERGIC-53 FP	GGCGTCTATGAGACAACACAGC
19	ERGIC-53 RP	GGTGGTAGTTCTGGGCATTTCG
20	TFG-FP	GAGAACCAGGACCTTCCACCAA
21	TFG-RP	GCTGCCATAACCTGAGTAGACTG
22	GAPDH FP	GTCTCCTCTGACTTCAACAGCG
23	GAPDH RP	ACCACCCTGTTGCTGTAGCCAA

24	HCV FP	CGGGAGAGCCATAGTGG
25	HCV RP	AGTACCACAAGGCCTTTCG
26	HCV<br>(probe)	CTGCGGAACCGGTGAGTACAC
27	GAPDH (probe)	CTCAAGATCATCAGCAATGCCTCCTGCAC
28	GAPDH FP	CATGAGAAGTATGACAACAGCC
29	GAPDH RP	TGAGTCCTTCCACGATACC
30	TFG cloning FP	TCAAGCTTGCCACCATGAACGGACAGTTGGATCTAAGTG
31	TFG cloning RP	TCCTCGAGTCGATAACCAGGTCCAGGTTGG
